# Cancer associated fibroblast derived SLIT2 drives gastric cancer cell metastasis by activating NEK9

**DOI:** 10.1038/s41419-023-05965-z

**Published:** 2023-07-13

**Authors:** Guofang Lu, Rui Du, Jiaqiang Dong, Yi Sun, Fenli Zhou, Fan Feng, Bin Feng, Ying Han, Yulong Shang

**Affiliations:** 1 State Key Laboratory of Cancer Biology and National Clinical Research Center for Digestive Diseases, Xijing Hospital of Digestive Diseases, Fourth Military Medical University, Xi’an, 710032 China; 2 Department of Physiology and Pathophysiology, National Key Discipline of Cell Biology, Fourth Military Medical University, Xi’an, 710032 China; 3 Institute for Biomedical Sciences of Pain, Tangdu Hospital, Fourth Military Medical University, Xi’an, 710038 China; 4 Department of Ultrasound Diagnostics, Tangdu Hospital, Fourth Military Medical University, Xi’an, 710038 China; 5 Department of Radiation Oncology, Xijing Hospital, Fourth Military Medical University, Xi’an, 710032 China

**Keywords:** Metastasis, Gastrointestinal cancer

## Abstract

The secretory properties of cancer-associated fibroblasts (CAFs) play predominant roles in shaping a pro-metastatic tumor microenvironment. The present study demonstrated that SLIT2, an axon guidance protein, produced by CAFs and promoted gastric cancer (GC) metastasis in two gastric cancer cell lines (AGS and MKN45) by binding to roundabout guidance receptor 1 (ROBO1). Mass-spectrometry analysis revealed that ROBO1 could interact with NEK9, a serine/threonine kinase. And their mutual binding activities were further enhanced by SLIT2. Domain analysis revealed the kinase domain of NEK9 was critical in its interaction with the intracellular domain (ICD) of ROBO1, and it also directly phosphorylated tripartite motif containing 28 (TRIM28) and cortactin (CTTN) in AGS and MKN45 cells. TRIM28 function as a transcriptional elongation factor, which directly facilitate CTTN activation. In addition, Bioinformatics analysis and experimental validation identified transcriptional regulation of STAT3 and NF-κB p100 by TRIM28, and a synergetic transcription of CTTN by STAT3 and NF-κB p100 was also observed in AGS and MKN45. Therefore, CAF-derived SLIT2 increased the expression and phosphorylation levels of CTTN, which induced cytoskeletal reorganization and GC cells metastasis. A simultaneous increase in the expression levels of NEK9, TRIM28 and CTTN was found in metastatic GC lesions compared with paired non-cancerous tissues and primary cancer lesions via IHC and Multiplex IHC. The analysis of the data from a cohort of patients with GC revealed that increased levels of NEK9, TRIM28 and CTTN were associated with a decreased overall survival rate. On the whole, these findings revealed the connections of CAFs and cancer cells through SLIT2/ROBO1 and inflammatory signaling, and the key molecules involved in this process may serve as potential biomarkers and therapeutic targets for GC.

## Introduction

Cancer initiation, progression and metastasis elicit a wide spectrum of dynamic alterations in host tissues, leading to the formation of a complex tumor stroma, also known as the tumor microenvironment (TME) [1, 2]. It is clear that the loss of the balanced activation and inactivation of genes associated with cytoskeletal remodeling is a key step of cancer metastasis. However, cancer progression and metastasis do not depend solely on cancer cell-autonomous defects, and the stimuli from the TME either trigger or enhance this process, creating a local microenvironment that enables cancer cells to acquire excessive motility, migrate to distant sites and form metastatic niches [1–6].

A dominant component of the TME is fibroblasts, and a number of studies over the years have suggested a prominent functional role for fibroblasts in cancer metastasis [1–8]. Quiescent or resting fibroblasts become activated in response to tissue damage as a consequence of neoplasia [1, 2]. The expression of α-smooth muscle actin (α-SMA) is the hallmark of activated fibroblasts. These cells may acquire further secretory phenotypes and robust autocrine activation, transforming into cancer-associated fibroblasts (CAFs), in the presence of persisting and unabated stress from the development of cancer lesions [2]. CAFs exert direct or indirect effects on cancer progression and tumor immunity through their secretome properties [1–6]. CAFs have been reported to secrete a wide range of growth factors, chemokines and cytokines, such as TGF-β, IL-6 and a series of CC chemokines (1 through 5). These factors can generate an enhanced inflammatory microenvironment and their binding to the receptors on the cancer cells also initiates the aberrant activation of metastasis-associated genes.

SLIT2 is an axon guidance protein that has recently been suggested to be secreted by CAFs [9–11]. Its binding to roundabout guidance receptor 1 (ROBO1) can affect a variety of downstream pathways, such as β-catenin/LEF/TCF, PI3K/Akt, CXCL12/CXCR4 and RAFTK/Pyk2, and can regulate cancer invasion and metastasis [12]. The authors’ laboratory has led several leading researches that have specifically clarified the upstream mechanisms of SLIT2/ROBO1 in gastric cancer (GC) metastasis [13, 14]. SLIT2 has been found to be associated with miR-218, which directly targets ROBO1, POU class 2 homeobox 2 (POU2F2) and IKK-β [13, 14]. In addition, IKK-β activates NF-κB and further promotes the transcriptional activation of POU2F2 by NF-κB [13, 14]. POU2F2 then continues to regulate ROBO1 through its transcriptional activities [13, 14]. ROBO1, POU2F2 and IKK-β then form a positive feedback loop and amplify the signaling from SLIT2 stimulation. As a transmembrane protein, ROBO1 binds to SLIT2 and acts on a series of signaling transduction mechanisms in targeted cells. Previous studies have demonstrated that ROBO1 can interact with a small group of Rho GTPase activating proteins [11, 15], and these proteins can directly affect the activation states of RhoA and Cdc42, thus leading to the reorganization of the cytoskeleton. This phenomenon appears to be fundamental to the regulation of cell motility by SLIT2/ROBO1; however, the spectrum of ROBO1-interacting proteins has not yet been fully explored. It also remains to be determined whether the SLIT2/ROBO pathway plays a role in the inflammatory microenvironment induced by cytokines derived from CAFs.

The present study analyzed the interacting proteins of ROBO1 using mass spectrometry (MS)-based analysis. It was found that NEK9, a serine/threonine kinase, which was found to an effector of IL-6 in a previous study by the authors [16], directly interacted with ROBO1, and fibroblast-derived SLIT2 further enhanced their mutual binding activities. The kinase domain of NEK9 was critical in its interaction with the intracellular domain (ICD) of ROBO1, and it also directly phosphorylated tripartite motif containing 28 (TRIM28) and cortactin (CTTN). TRIM28 function as a transcriptional elongation factor, which directly facilitate CTTN activation. In addition, bioinformatics analysis and experimental validation identified transcriptional regulation of STAT3 and NF-κB p100 by TRIM28, and a synergetic transcription of CTTN by STAT3 and NF-κB p100 was also observed in AGS and MKN45. Therefore, CAFs-derived SLIT2 increased the expression and phosphorylation levels of CTTN, which induced cytoskeletal reorganization and led to the acquirement of excessive migratory capacities of cancer cells. The findings presented herein reveal the associations between CAFs and cancer cells through SLIT2/ROBO1 and inflammatory signaling, and the key molecules involved may serve as potential biomarkers and therapeutic targets for GC.

## Material and methods

### Cells and cell culture

The human gastric cancer lines, AGS, were purchased from the China Infrastructure of Cell Line Resources in September, 2013. Human MKN45 cell line was kindly provided by Dr. Zhao S (State Key Laboratory of Cancer Biology and National Clinical Research Center for Digestive Diseases, Xijing Hospital of Digestive Diseases, Fourth Military Medical University, Xi’an, China). The cells were cultured in RPMI-1640 medium with 10% FBS (South America origin; IC-1905; BioCytoSci) and 100 μg/ml penicillin-streptomycin at 37 °C in a 5% CO_2_ incubator.

### Extraction of CAFs

Post-operative gastric cancer tissue was transferred to a biological safety cabinet, chopped and washed three times in phosphate-buffered saline (PBS). The tissues were digested with 0.1% collagenase type I, II and IV and 0.1% dispase at 4 °C overnight. The following day, the tissue and digestion solution were centrifuged (12000 x g), and the supernatant was discarded. This was followed by the addition of 0.1% type II collagenase for digestion in a water bath at 37 °C for 2–3 h. The contents were then passed through a 100-mesh sieve and centrifuged at 300 x g for 5 min at RT. Finally, the cells were resuspended using appropriate medium at 37 °C in a 5% CO_2_ incubator.

### Plasmid construction and transfection

The construction of all plasmids, including STAT3, p100 and the CTTN promoter was performed according to the manufacturer’s procedures as outlined in the authors laboratory and as previously described [17]. The sequences are presented in Tables S2–S4. The overexpression or knockdown of TRIM28, CTTN plasmids, and knockdown of SLIT2 plasmids, containing single or mutation phosphorylation site, were obtained from Shanghai Genechem Co., Ltd. The plasmids were transfected into the cells using Lipofectamine™ 2000^®^ (Invitrogen; Thermo Fisher Scientific, Inc.) and GP-transfect-mate (Shanghai Genechem Co., Ltd.) according to the standard instructions and as previously described [16].

### Immunohistochemistry (IHC)

Briefly, the sections were dewaxed in xylene and rehydrated in ethanol and PBS. Endogenous peroxidase was inactivated using 3% H_2_O_2_, and antigen retrieval was performed using the Tris EDTA antigen retrieval buffer for TRIM28 staining and Citrate Antigen Retrieval Solution for NEK9 and CTTN staining. The sections were blocked with 10% normal goat serum at room temperature for 15 min and incubated with primary antibodies against TRIM28 (cat. no. sc-515790, Santa Cruz Biotechnology, Inc.), NEK9 (cat. no. NBP2-31091, Novus Biologicals, LLC) and CTTN (cat. no. sc-55579, Santa Cruz Biotechnology, Inc.) at 4 °C overnight. The sections were then incubated with corresponding secondary antibodies conjugated with horseradish peroxidase at room temperature for 30 min. Staining was visualized using a DAB kit (Zhongshan Golden Bridge Biotechnology). All sections were examined and scored independently by two investigators in a double-blinded manner. Staining and the score were determined as previously described [16, 18].

### Western blot analysis

Total protein was extracted using RIPA lysis buffer containing protease and phosphatase inhibitor cocktail, EDTA-free, 100X (APT006 or APT008, AntiProtech Inc.) Western blot analysis was performed using standard procedures as previously described [18]. The antibodies used were as follows: anti-NEK9 (cat. no. ab138488, Abcam), anti-ROBO1 (cat. no. 20219-1-AP, ProteinTech Group, Inc.), anti-Flag (cat. no. MA5558, AntiProtech Inc.), anti-STAT3 (cat. no. 12640, Cell Signaling Technology, Inc.), anti-p-STAT3 (cat. no. 9145, Cell Signaling Technology, Inc), anti-mouse IgG H&L (Alexa Fluor 488) (cat. no. ab150113, Abcam), anti-TRIM28 (cat. no. 15202-1-AP, ProteinTech Group, Inc.), anti-CTTN (cat. no. sc-55579, Santa Cruz Biotechnology, Inc.) anti-pCTTN (cat. no. 4569, Cell Signaling Technology, Inc.), anti-p100 (cat. no. 4882, Cell Signaling Technology, Inc.), anti-HA (cat. no. 51064-2-AP, ProteinTech Group, Inc.), anti-p-Ser (cat. no. sc-81514, Santa Cruz Biotechnology, Inc.) and anti-β-actin (cat. no. A2228, clone AC-74, Millipore Sigma).

### In vivo model of metastasis and bioluminescence imaging

BALB/C nude mice (6 weeks old) were obtained from Vital River Laboratories (Beijing, China), and cared for according to the institutional guidelines for animal care. Animals were randomly allocated to control groups and metastasis model groups (*n* = 10). For the in vivo model of metastasis, 3 × 10^6^ cells in PBS were injected into the tail veins of the nude mice. After 8 weeks, the mice were intraperitoneally injected with D-luciferin (Caliper Life Sciences), and the mice were anesthetized using inhalant anesthetics (2–5% isoflurane for 5 min). Subsequently, whole-body live images were captured using IVIS Imaging System (Xenogen) software. As the strong signals from the orthotopic tumors masked the much weaker lung and liver metastatic signals, the mice were then immediately sacrificed by cervical dislocation under deep anesthesia, and whole lungs and livers were harvested for the detection of bioluminescence signals, and lungs and livers were dissected and prepared for use in standard hematoxylin and eosin (H&E) staining. All tissues were dissected and staining independently by two investigators in a double-blinded manner.

### In vitro migration and invasion assay

For Transwell assays, 4 × 10^4^ cells in each group in 200 μl serum-free medium were seeded into the upper chamber of the Transwell (8.0 μm pore, Corning, Inc.) without (migration) or with (invasion) Matrigel (BD Biosciences). The lower chamber was filled with RPIM-1640 (600 μl) medium containing 20% FBS. A 4% polymethanol solution was used to fix the cells in the upper chambers after 24 h of seeding, and crystal violet was used to stain (RT 10 mins) the cells. Images were captured at x 200 magnification using an Olympus BX51 microscope (Olympus Corporation). A total of five random fields were selected and the number of cells were counted for migration or invasion.

### Immunofluorescence (IF) and cytoskeletal staining

Following fixation with 4% paraformaldehyde in PBS for 10 min, the cells were washed twice with PBS. The GC cells were incubated with the primary antibodies anti-NEK9 (cat. no. sc-100401, Santa Cruz Biotechnology, Inc. for IP and IF), anti-CTTN (cat. no. sc-55579, Santa Cruz Biotechnology, Inc.), anti-SLIT2 (cat. no. 20217-1-AP, ProteinTech Group, Inc.), anti-α-SMA (cat. no. GB111364, Servicebio) and anti-α-tubulin (cat. no. ab7291, Abcam) overnight at 4 °C and the secondary antibody anti-mouse IgG H&L (Alexa Fluor 488) (ab150113, Abcam) 1 h at room temperature. Finally, the cells were washed and mounted with mounting medium containing DAPI (Vector Laboratories, Inc.). For phalloidin staining, the GC cells were incubated with phalloidin for 40 min at room temperature. Images were captured using a Nikon A1 Plus confocal microscope (Nikon Corporation).

### In vitro kinase assay

The phosphorylation of TRIM28 and CTTN (human) Recombinant Protein (P01) (H00010155-P01, H00002017-P01, Abnova) by active NEK9 protein (1ug/ml, ab125614, Abcam) was monitored using a Universal Kinase Activity Kit (R&D Systems, Inc.) according to manufacturer’s instructions. As described in a previous study by the authors, the optical density (OD) at 620 nm was used to determine the amount of phosphorylated TRIM28 and CTTN [16].

### Statistical analysis

All analyses were performed using GraphPad Prism version 9.4.0 for Windows, GraphPad Software, San Diego, California USA, www.graphpad.com. The data are expressed as the mean ± SEM. Differences between two groups were assessed using Mann-Whitney *U* test, while one-way ANOVA (and Kruskal-Wallis *H* tests) was used for comparing multiple groups. The survival rates were determined using the Kaplan-Meier method and the log-rank test. Correlations were analyzed using Spearman’s correlation analysis based on the normality test (*r*_*s*_, *P*-value). A value of *P* < 0.05 was considered to indicate a statistically significant difference.

## Results

### Binding of CAF- SLIT2 to ROBO1 promotes GC metastasis and cytoskeletal reorganization

**Fig. 1:**
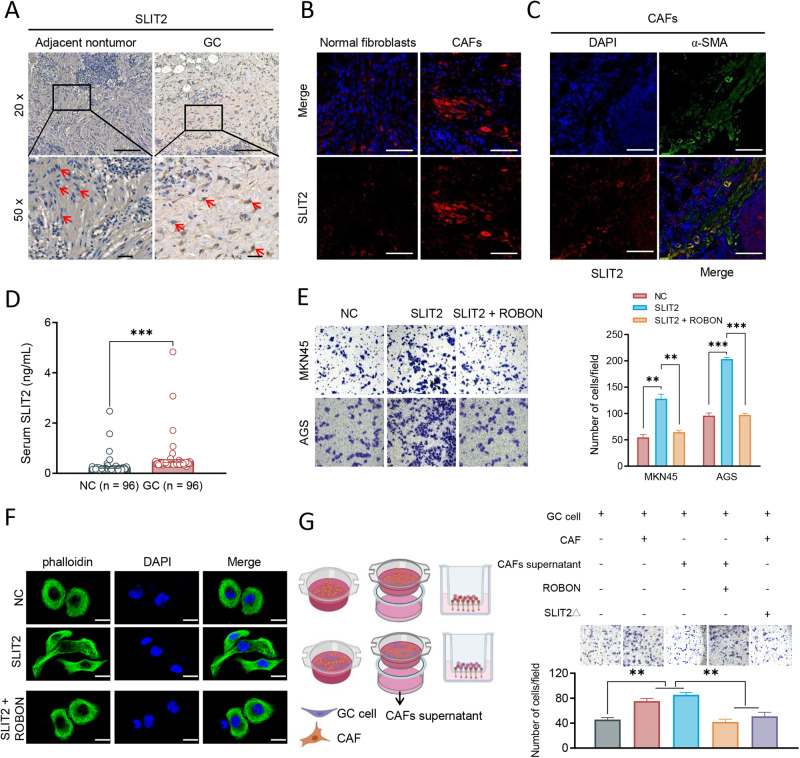
CAF SLIT2 promotes GC metastasis and cytoskeletal reorganization in a ROBO1-dependent manner. **A** Representative images of IHC staining for SLIT2 in adjacent nontumor and CAFs in GC tissues. Long scale, 50 μm, short scale, 20 μm. **B** Representative images of IF staining for SLIT2 level in Normal fibroblasts and CAFs cells. **C** Representative images of IF staining for SLIT2 (red) and α-SMA (green) in CAFs of GC tissues. (**D**) SLIT2 levels in serum from healthy volunteers (*n* = 96) and gastric cancer patients via ELISA (*n* = 96). **E** SLIT2 promoted GC MKN45 and AGS cell migration, ROBON treatment inhibited cell migration by in vitro transwell analysis, SLIT2, recombinant, 100 ng/ ml, 24 hours, ROBON, a soluble ROBO1 receptor antagonist. **F** Representative images of cytoskeleton staining with phalloidin for 40 min at room temperature for SLIT2 treatment in GC cells, Scale, 50 μm. **G** Left panel represents the co-culture pattern diagram in human gastric cancer cells, CAFs and CAF culture supernatant. Right panel demonstrates either CAFs or their culture supernatant can promote GC cell migration, while ROBON and SLIT2 knockdown in CAFs antagonizes cell migration by in vitro transwell analysis. ^**^P < 0.01 and ^***^P < 0.001. CAF cancer-associated fibroblasts, GC gastric cancer, ROBO1, roundabout guidance receptor 1, SLIT2Δ, SLIT2 knockdown in CAFs.

The expression and location of SLIT2 were examined using IHC and IF staining in normal fibroblasts and CAFs in GC tissues. An increased SLIT2 were detected in CAFs (red arrows) of GC tissues compared to adjacent nontumor tissue (Fig. 1A) by IHC. Compared with the normal fibroblasts cell, SLIT2 expression was increased in CAFs in GC tissues (Fig. 1B). α-SMA was used to mark CAFs and the co-immunostaining of α-SMA and SLIT2 was confirmed (Fig. 1C), suggesting that SLIT2 was secreted by CAFs. The serum levels of SLIT2 (*n* = 96) were detected using ELISA, the results revealed a notable increase in SLIT2 levels in patients with GC (Fig. 1D). And the recombinant SLIT2 protein treatment (100 ng/ ml, 24 hours) enhanced the migratory ability of the MKN45 and AGS cells (Fig. 1E). A regular cell shape and actin stress fibers were observed in GC cells using cytoskeletal staining, and SLIT2 induced a transition to a spindle-like shape with the formation of lamellipodia (Fig. 1F). All these effects of SLIT2 on GC cells were blocked by ROBON, a soluble ROBO1 receptor antagonist, suggesting that the SLIT2 promoted GC metastasis in a ROBO1-dependent manner. Either CAFs or their culture supernatant promoted GC cell migration in vitro, ROBON and SLIT2 knockdown in CAFs antagonized this effect (Fig. 1G), further confirming the pro-metastatic roles of SLIT2/ROBO1 in GC.

### SLIT2 enhances the interaction of ROBO1 and NEK9

**Fig. 2:**
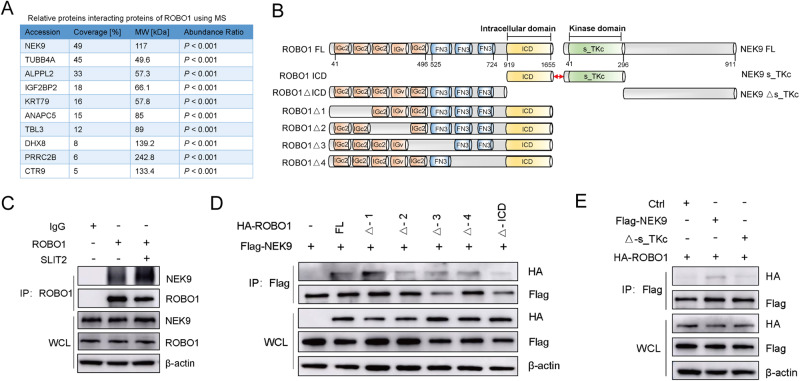
SLIT2 increases the interaction of ROBO1 and NEK9 in gastric cancer cells. **A** Mass-spectrometry analysis was used to predict the interacting proteins of ROBO1. **B** The protein domain structure diagram for ROBO1 Ig domains, three fibronectin III repeats, intracellular domain and NEK9 kinase domain were predicted by SMART online database. **C** Co-immunoprecipitation (Co-IP) assay demonstrated the interaction between endogenous ROBO1 and NEK9 in GC cells, and SLIT2 treatment (100 ng/ ml, 24 hours) enhanced this interaction. **D** The GC cell lysates co-transfected with Flag-NEK9 and HA-ROBO1 (the full-length or deletion mutants) were used to detect the interaction using Co-IP assay. **E** Co-IP assay demonstrating the interaction between ROBO1 and mutation kinase domain of NEK9 in GC cells. ROBO1, roundabout guidance receptor 1.

To further explore the mechanisms of SLIT2/ROBO1, the potential interacting proteins of ROBO1 were analyzed using Mass-spectrometry (MS). As shown in Fig. 2A, NEK9 was found to be the top candidate protein. The protein structure of ROBO1 and NEK9 were predicted using SMART online software (https://smart.embl.de/) [19], full-length and series of truncated ROBO1 and NEK9 plasmids were constructed (Fig. 2B). A direct interaction of endogenous ROBO1 and NEK9 was validated using Co-IP, and pre-treatment with SLIT2 (100 ng/ ml, 24 hours) induced a significantly stronger binding of ROBO1 and NEK9 (Fig. 2C). The intracellular domain (ICD) of ROBO1 appeared to be critical for its interaction with NEK9, as the deletion of the ICD blocked their interaction, while the deletion of other domains only had a minimal effect (Fig. 2D). In addition, NEK9 lacking the kinase domain could not interact with ROBO1 (Fig. 2E). On the whole, these results demonstrated that the ICD of ROBO1 interacted with kinase domain of NEK9, and the recombinant SLIT2 treatment enhanced their mutual binding activities.

### NEK9 regulates cell motility by phosphorylating TRIM28 and CTTN

**Fig. 3:**
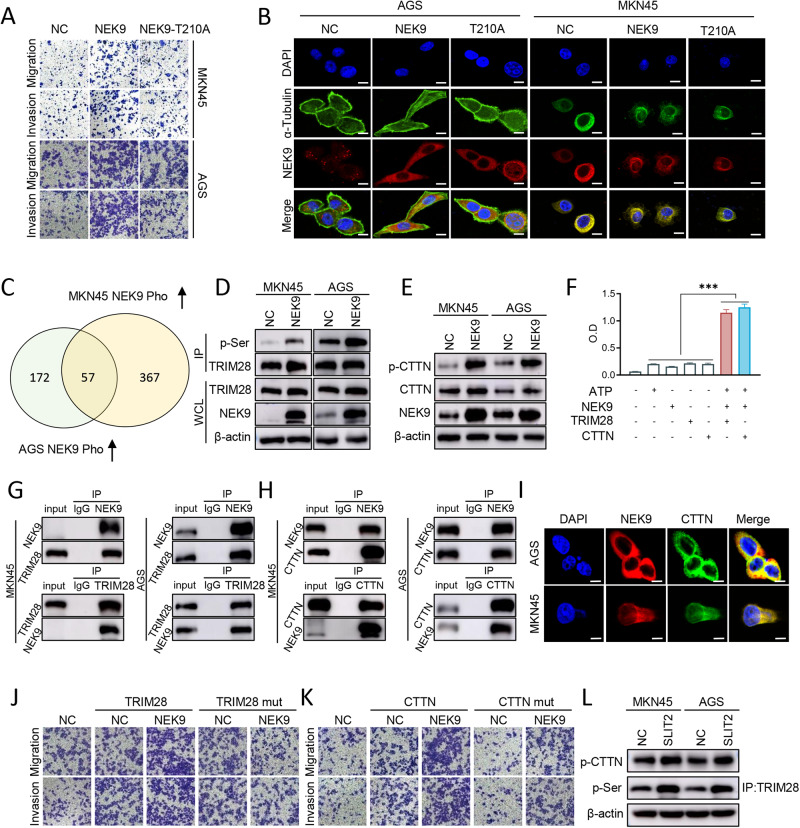
NEK9 regulates gastric cancer cell migration and cytoskeletal reorganization by phosphorylating TRIM28 and CTTN. **A** AGS and MKN45 cells were transfected NEK9 overexpression and mutant NEK9 (kinase-dead mutant NEK9) plasmid, and the cell migration ability was evaluated via in vitro Transwell assay. **B** Representative images of IF staining for NEK9 (red) and α-tubulin (green) in AGS and MKN45 cell for cell shape and lamellipodia formation, Scale, 20 μm. **C** Phosphoproteomics analyses with stable AGS and MKN45 NEK9-overexpression transfection was used to screen the 57 candidate effector proteins of NEK9. **D** and **E** Western blot analysis for the phosphorylation levels of TRIM28 (**D**) and CTTN (**E**) in AGS and MKN45 cells with NEK9 overexpression treatment. **F** NEK9 was found to phosphorylate TRIM28 and CTTN using an in vitro kinase assay. **G** and **H** Co-immunoprecipitation assay illustrating the interaction between NEK9 and TRIM28 (**G**), and NEK9 and CTTN (**H**) in AGS and MKN45 cells. **I** Representative images of IF staining for NEK9 (red) and CTTN (green), showing the colocalization in AGS and MKN45 cell, Scale, 20 μm. **J** and **K** The function of NEK9, TRIM28, TRIM28 mutation, CTTN and CTTN mutation on cell motility were evaluated via in vitro Transwell assay. **L** The phosphorylation levels of TRIM28 and CTTN were assessed with SLIT2 treatment (100 ng/ ml, 24 hours) in AGS and MKN45 cells via Western blot analysis. TRIM28 mut: mutant TRIM28 (S473 and S824), CTTN mut: mutant CTTN (S405, S417, S418 and S447), ^***^P < 0.001. TRIM28 tripartite motif containing 28, CTTN cortactin.

A previous study by the authors confirmed that NEK9 functions as an effector of IL-6/STAT3 by targeting ARHGEF2 phosphorylation [16]. To assess whether NEK9 induces the GC cells metastasis depending on its kinase activity, the current study compared the effects of wild-type NEK9 (NEK9-WT) and kinase-dead mutant NEK9 (NEK9-T210A) on GC cells. It was found that NEK9 promoted GC cell motility in vitro (Fig. 3A), and it also induced cytoskeletal reorganization, manifested as changes in cell shape and lamellipodia formation (Fig. 3B). All these effects were attenuated by a T210A mutation of NEK9. Subsequently, phosphoproteomics analysis was performed using AGS and MKN45 cells stably overexpressing NEK9 transfection, and 57 candidate target proteins of NEK9 were found (Fig. 3C, Table S1). Among these proteins, 11 proteins were associated with the cytoskeleton and inflammation. Furthermore, clinical analyses of the data from The Cancer Genome Atlas (TCGA) and KM plotter databases were performed, and found that TRIM28 and CTTN expression levels were increased in GC and associated with a decreased survival rate (Fig. S1). An increase in the serine phosphorylation of TRIM28 by NEK9 was observed, and it also increased the total and phosphorylated protein levels of CTTN (Fig. 3D, E). In addition, in vitro kinase assay indicated that TRIM28 and CTTN were the direct targets of NEK9 (Fig. 3F). The direct interactions and co-localization of NEK9 with its substrates were validated using Co-IP and IF staining (Fig. 3G–I). Moreover, mutations of the phosphorylation sites of TRIM28 (mutant of S473 and S824) and CTTN (mutant of S405, S417, S418 and S447) not only blocked their pro-metastasis effects on GC cells, but also attenuated the effects of NEK9 (Fig. 3J, K). The phosphorylation levels of TRIM28 and CTTN were assessed with SLIT2 treatment (100 ng/ ml, 24 hours) in AGS and MKN45 cells, the phosphorylation expression of TRIM28 and CTTN was upregulated compared with the NC group (Fig. 3L).

**Fig. 4:**
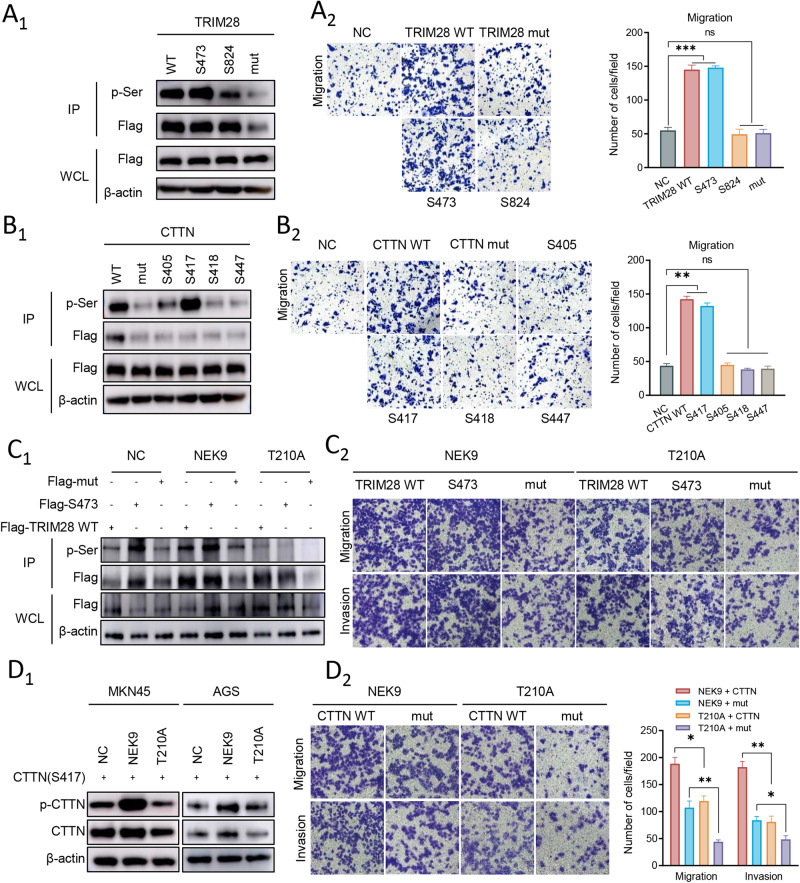
NEK9 phosphorylates TRIM28 and CTTN on the S473 and S417 phosphorylation site, separately. **A, B** The potential functional serine residue of TRIM28 and CTTN, the wild-type, single-site wild-type TRIM28 (S473 and S824) mutant, TRIM28 mut (mutant S473 and S824), single-site wild-type CTTN (S405, S417, S418 and S447) and CTTN mut (mutant S405, S417, S418 and S447) constructs were separately transfected into AGS cells to detect the expression level of serine residue and cell migration using (A_1_, B_1_) Co-IP and (A_2_, B_2_) in vitro Transwell assay. **C** and **D** Gastric cancer cells were transfected NEK9 overexpression and NEK9-T210A plasmid to evaluated the TRIM28 (**C**) and CTTN (**D**) phosphorylation level and cell motility abilities using Co-IP, western blot analysis and in vitro cell migration assay. ^*^P < 0.05 and ^**^P < 0.01.TRIM28 tripartite motif containing 28, CTTN, cortactin.

To clear the potential functional serine residue of TRIM28 and CTTN, the wild-type, mutant and single-site wild-type TRIM28 (S473 and S824) and single-site wild-type CTTN (S405, S417, S418 and S447) constructs were separately transfected into AGS cells. The phosphorylation levels of S473 and S824 and cell migration functions were detected, and only phosphorylation on S473 exerted similar effects as wild-type TRIM28 on cell motility (Fig. 4A). Similarly, the results revealed only phosphorylation on S417 exerted similar effects as wild-type CTTN on cell motility (Fig. 4B). The results of western blot analysis and in vitro cell migration assay revealed that NEK9-T210A did not induce TRIM28 phosphorylation and cell motility in vitro (Fig. 4C). Similar results were also found in CTTN and its phosphorylation mutant (Fig. 4D). Overall, these findings suggested that the phosphorylation of TRIM28 and CTTN by NEK9 was a key step in NEK9-induced GC metastasis and cytoskeletal reorganization.

### TRIM28 induces cytoskeletal reorganization and cell migration by transactivating CTTN

The potential roles of TRIM28 and CTTN in GC were further investigated in a series of in vitro functional assays. As shown in Figs. S2, S3, the overexpression of TRIM28 and CTTN increased the motility of MKN45 and AGS cells, whereas the knockdown of TRIM28 and CTTN inhibited this phenomenon. These findings indicated that TRIM28 and CTTN played oncogenic roles in GC metastasis.

**Fig. 5:**
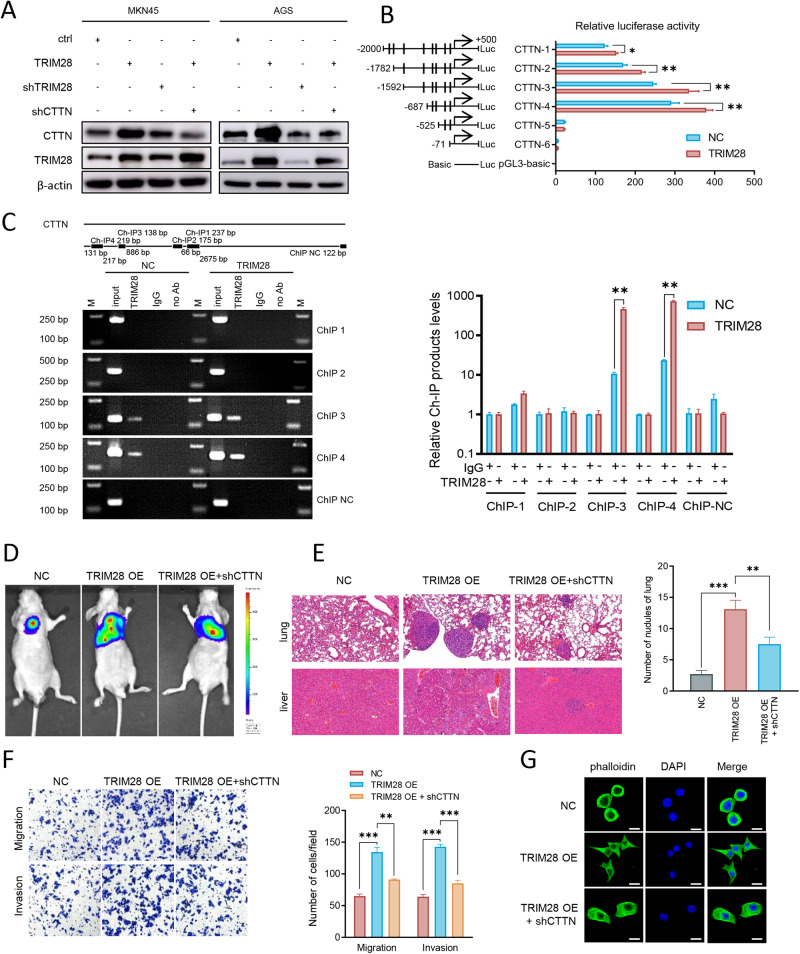
TRIM28 induces cytoskeletal reorganization and cell migration through the transactivation of CTTN expression. **A** AGS and MKN45 cells were transfected TRIM28, shTRIM28 and shCTTN to identify the expression of TRIM28 and CTTN using Western blot analysis. **B** Serially truncated CTTN promoter constructs were cloned into pGL3-luciferase reporter plasmids and transfected into GC cells to calculate the relative luciferase activity. **C** ChIP and reverse transcription-quantitative PCR demonstrated the direct binding of TRIM28 to the CTTN promoter in GC cells. M, marker. **D** and **E** The in vivo metastatic assay revealed TRIM28 exerted a pro-metastatic effect on gastric cancer cells, and CTTN knockdown attenuated this effect using (**D**) bioluminescent imaging and (E) hematoxylin and eosin staining. **F** MKN45 cells were transfected TRIM28 overexpression and shCTTN to calculate the migration and invasion abilities by in vitro Transwell assay. **G** Cytoskeleton staining with phalloidin for 40 min at room temperature for TRIM28 and shCTTN treatment in GC cells, Scale, 20 μm. ^*^P < 0.05, ^**^P < 0.01 and ^***^P < 0.001. TRIM28 tripartite motif containing 28, CTTN, cortactin.

As described in Fig. 3E, an increase in CTTN was found following the transfection with NEK9 overexpression plasmid. Considering that TRIM28 can function through its transcriptional activities, it was hypothesized that the phosphorylation of TRIM28 may enhance the transcription of CTTN. Western blot analysis revealed that the upregulation of TRIM28 in MKN45 and AGS cells resulted in the increased expression of CTTN, and its knockdown inhibited CTTN expression. The knockdown of CTTN antagonized this effect (Fig. 5A). Subsequently, six putative TRIM28 binding sites were found in the CTTN promoter using the Jasper database, and a series of truncations were generated (Table S2). Compared with the control group, a deletion from nt-687 to nt-525 completely blocked TRIM28-induced CTTN promoter activity (Fig. 5B). Consistently, ChIP analysis confirmed the association of TRIM28 with the CTTN promoter (Fig. 5C). The in vivo metastatic assay revealed that TRIM28 exerted a pro-metastatic effect on GC, as its overexpression increased the incidence of lung and liver metastases (Fig. 5D, E). The knockdown of CTTN then attenuated this effect (Fig. 5D, E). Similarly, the in vitro cell migration assay and IF staining revealed that the knockdown of CTTN significantly reduced TRIM28-mediated cell migration and lamellipodia formation (Fig. 5F, G). These results suggested that TRIM28 induced cytoskeleton reorganization and cell migration by transactivating CTTN.

### STAT3 and NF-κB2 (p100) are the transcriptional targets of TRIM28, and both can transcriptionally activate CTTN

**Fig. 6:**
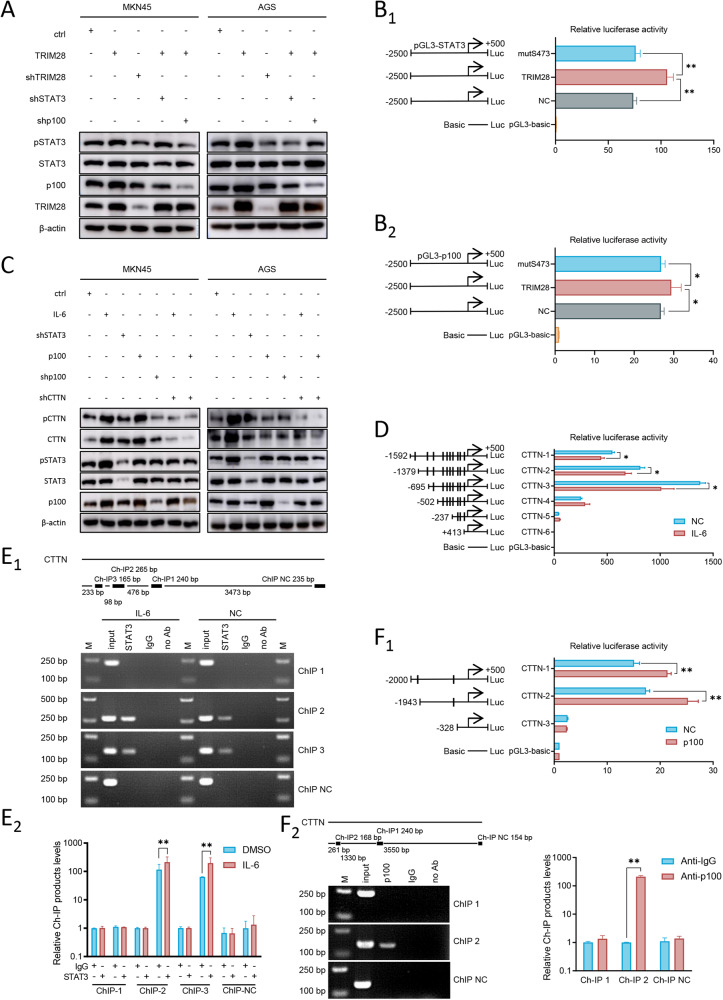
TRIM28 expression promotes CTTN expression and gastric cancer cell metastasis by the transactivation of STAT3 and NF-κB2 (p100). **A** MKN45 and AGS cells were transfected by TRIM28 overexpression, shTRIM28, STAT3 overexpression, shSTAT3, shp100 or the control. The expression levels of TRIM28, p100, STAT3 and p-STAT3 were detected using western blot analysis. (**B**_**1**_) STAT3 and (**B**_**2**_) p100 promoter constructs were cloned into pGL3-luciferase reporter plasmids and transfected into GC cells to calculate the relative luciferase activity of TRIM28 and mutation on S473. **C** MKN45 and AGS cells were transfected by IL-6 recombinant, shSTAT3, p100 overexpression, shp100, CTTN overexpression, shCTTN or the control. The expression levels of p100, STAT3, p-STAT3, CTTN and pCTTN were detected using western blot analysis. **D** Serially truncated CTTN promoter constructs were cloned into pGL3-luciferase reporter plasmids and transfected into GC cells to calculate the relative luciferase activity with IL-6 treatment, IL-6 (50 ng/ml, 10 min). **F**_**1**_ Serially truncated CTTN promoter constructs were cloned into pGL3-luciferase reporter plasmids and transfected into GC cells to calculate the relative luciferase activity with p100 overexpression. (**E** and **F**_**2**_) ChIP and reverse transcription-quantitative PCR assay demonstrated the direct binding of (**E**) STAT3 and (**F**_**2**_) p100 to the CTTN promoter in GC cells. M, marker. ^*^P < 0.05 and ^**^P < 0.01^.^ TRIM28 tripartite motif containing 28, CTTN, cortactin.

Of note, the present study found that there were also potential binding sites of STAT3 and NF-κB2 (p100) in the promoter region of CTTN. TRIM28 was first identified as a STAT-binding partner [20] and frequent crosstalk between IL-6/STAT3 and NF-κB has been observed in cancer progression [21]. Therefore, the present study wished to determine whether TRIM28 is interconnected with STAT3 and p100. A positive association between the TRIM28 expression level and STAT3 and p100 was confirmed using western blot analysis and TCGA data analysis (Figs. 6A and S4). Luciferase reporter assays revealed that TRIM28 could bind to the p100 or STAT3 promoter; however, the mutation on S473 completely blocked this binding (Fig. 6B), suggesting that phosphorylation on S473 was critical to the transcription activities of TRIM28.

To validate whether STAT3 and p100 also transcriptionally activate CTTN, western blot analysis and TCGA data analysis were used to reveal the positive association between TRIM28, STAT3 and p100 (Figs. 6C and S4). A series of reporters containing different binding sites was then co-transfected with STAT3 (Table S3), and IL-6-activated STAT3 could bind to the CTTN promoter between nt-1592 to nt-502 (Fig. 6D). Three potential binding sites were present in this region and the ChIP assay revealed that the site-2 and site-3 regions were the exact binding sites of STAT3 (Fig. 6E). The binding of p100 to the CTTN promoter was examined in similar experiments and it was found that p100 transactivated CTTN by binding to the site-2 region in the CTTN promoter (Table S4, and Fig. 6F). Taken together, TRIM28 could transactivate STAT3 and p100, and a synergetic transcription of CTTN by TRIM28, STAT3 and p100 was also found.

### NEK9, TRIM28 and CTTN are simultaneously upregulated in GC metastasis, and are associated with the prognosis and diagnosis of patients with GC

**Fig. 7:**
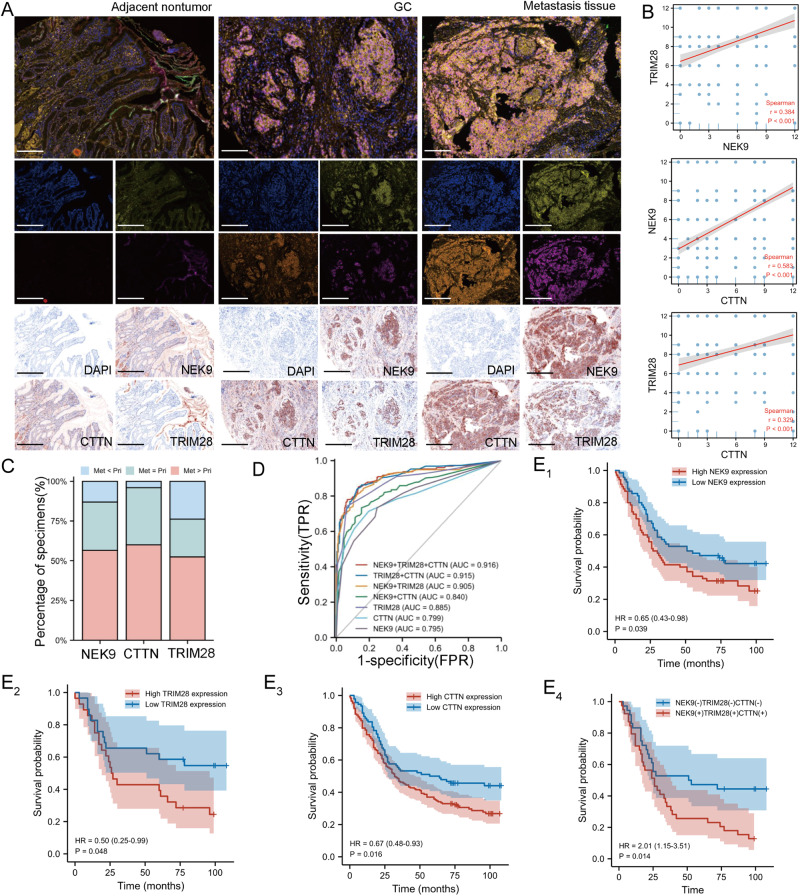
Simultaneous elevation of NEK9, TRIM28 and CTTN in metastatic GC. **A, C** Representative images of IHC and multiplex IHC staining for NEK9, TRIM28 and CTTN in adjacent non-tumor and gastric cancer tissues, Scale, 50 μm (**A**). **C** An increase in these proteins was found in GC metastases. **B** Positive association between the level of NEK9, TRIM28 and CTTN in GC. **D** Diagnostic value in patients with GC using receiver operating characteristic curve analysis. **E** Kaplan-Meier curves of patients with GC with low vs. a high expression of NEK9 (**E**_**1**_), TRIM28 (**E**_**2**_), CTTN (**E**_**3**_) and these three proteins (**E**_**4**_). TRIM28, tripartite motif containing 28; CTTN cortactin; IHC immunohistochemistry; GC gastric cancer.

To evaluate the clinical significance of NEK9, TRIM28 and CTTN in patients with GC, their expression levels were assessed in a series of tissue microarrays using IHC and multiplex IHC. The results confirmed that the expression levels of NEK9, TRIM28 and CTTN were higher in primary GC tissues than adjacent noncancerous tissues (Fig. 7A and Fig. S5A). The staining intensity of NEK9, TRIM28 and CTTN was also significantly higher in distant metastatic lesions than that in primary tissues (Fig. 7A, C). In GC cases with clinicopathological information, the intensities of NEK9, TRIM28 and CTTN were associated with pathological stage and T/N staging (Tables S5–S7). Additionally, a positive association between NEK9, TRIM28 and CTTN was detected (Fig. 7B), which was in agreement with the results obtained using the data from TCGA (Fig. S5B). Subsequently, receiver operating characteristic (ROC) curve analysis of NEK9, TRIM28 and CTTN was conducted to evaluate the diagnostic value in patients with GC using the R package pROC (Version 3.6.3). The area under the curve (AUC) of single NEK9, TRIM28 and CTTN was 0.795, 0.885 and 0.799, respectively; the AUC of NEK9 plus CTTN, NEK9 plus TRIM28, and TRIM28 plus CTTN was 0.840, 0.905, 0.915, respectively. It is worth noting that the AUC of all three gene was 0.916, which was significantly higher than the other grouping methods (Fig. 7D). Patients with higher expression levels of NEK9, TRIM28 and CTTN exhibited a reduced overall survival rate, and a combination of these proteins could better distinguish patients with different survival rates (Fig. 7E). Overall, these findings suggested the potential roles of this pathway in the evaluation of the metastatic potential and prognosis of patients with GC.

**Fig. 8:**
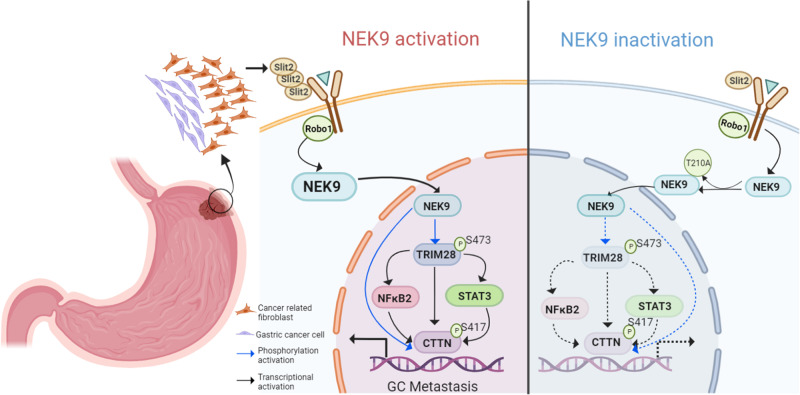
Model of the SLIT2/ROBO1/NEK9 signaling pathway in gastric cancer. NEK9 kinase domain and the intracellular domain of ROBO1 was enhanced by cancer-associated fibroblast-derived SLIT2. NEK9 directly phosphorylated TRIM28 (S473) and CTTN (S417). Phosphorylation on S473 of TRIM28 increased its transcriptional activities on STAT3, p100 and CTTN. TRIM28, STAT3 and p100 functioned synergistically to transcriptionally activate CTTN. Increased expression and phosphorylation levels of CTTN led to cytoskeletal reorganization and gastric cancer metastasis. ROBO1, roundabout guidance receptor 1; TRIM28, tripartite motif containing 28; CTTN, cortactin.

In summary, the present study reported a new molecular mechanism of SLIT2 in GC cytoskeletal reorganization and metastasis. As shown in Fig. 8, CAF-derived SLIT2 enhanced the direct interaction between the ICD of ROBO1 and NEK9 kinase domain. NEK9 directly phosphorylated TRIM28 and CTTN. The phosphorylation of TRIM28 on S473 increased it transcriptional activities on STAT3, p100 and CTTN. In addition, a synergetic transcription of CTTN by TRIM28, STAT3 and p100 was also found. The increased expression and phosphorylation levels of CTTN led to cytoskeletal reorganization and GC metastasis.

## Discussion

In the present study, a novel SLIT2/ROBO1/NEK9 pathway was identified that bridged CAF and GC metastasis. CAF-derived SLIT2 promoted the mutual interaction between the ICD of ROBO1 and NEK9, and an increase in the protein function of TRIM28 and CTTN, two target proteins phosphorylated by NEK9, was observed in GC metastasis. Phosphorylated TRIM28 enhanced its transactivation of CTTN, STAT3 and NF-κB p100, and CTTN was also the transcriptional target of STAT3 and NF-κB p100. These findings demonstrated that CAF-derived SLIT2 increased the expression and function activity of CTTN by NEK9, which enabled GC cells to acquire excessive migratory capacities through cytoskeletal remodeling.

Compared with quiescent or resting forms, activated fibroblasts are more morphologically and metabolically active, and their dynamic secretory properties are also critical in cell-cell communications [2]. The soluble proteins factors affect tumor cell motility and metastasis. Growth factors and cytokines, such as TGF-β1 and SDF-1 not only exert profound effects on cancer cell motility, but also promote the activation of CAFs [3]. Herein, it was found that CAF-derived SLIT2 promoted GC metastasis, which was consistent with the findings of previous studies [13, 14]. The knockdown of SLIT2 has also been shown to inhibit glioblastoma progression, suggesting its oncogenic roles in cancer [22]. However, the inhibitory effects of SLIT2 on cancer progression and metastasis have also been reported in pancreatic cancer, lung cancer, breast cancer and thyroid cancer [9, 15, 23–27]. It is not surprising that the role of SLIT2 is dependent on cell origins and context, thus promoting SLIT2 to function in a cell type-specific manner. In addition, the deletion of endothelial SLIT2 has been shown to suppress metastatic dissemination in mouse models of breast and lung cancer, while the deletion of tumoral SLIT2 enhances metastatic progression [28]. These findings suggest that endogenous and exogenous SLIT2 may play distinctive roles in regulating cancer cell behaviors. The present study focused on SLIT2 from CAFs, and this partially explained the differences between the findings presented herein and other conclusions drawn from other research on tumoral SLIT2.

In addition, even fibroblast-derived SLIT2 also exerts opposite effects in different types of cancer [9, 10], and this phenomenon is probably associated with the biological heterogeneity and versatility of CAFs [1, 2]. There are dynamic and interchangeable shifts of individual classes of CAFs between either tumor-promoting or tumor-restraining phenotypes, and this process is mainly dependent on the complex context of the surrounding TME [1, 2]. The secretory factors of CAFs, either in soluble form or exosomes, also communicate with innate and adaptive immune cells in the TME [1, 2, 5, 6]. A combination of these factors, as well as cancer cells determines whether metastasis will occur or not. The findings of the present study suggested that targeting CAF-derived SLIT2 may be a potential therapeutic target in GC. Considering that it was difficult to distinguish CAFs with different phenotypes due to a lack of definitive biomarkers and signaling pathways, it was more practical to target SLIT2/ROBO1, as the blocking of SLIT2 binding to ROBO1 by a soluble ROBO1 antagonist significantly attenuated SLIT2-induced GC metastasis.

The interaction between NEK9 and ROBO1 appears to be crucial for SLIT2/ROBO1 signaling transduction. The kinase domain directly interacts with ROBO1, a process that is enhanced by SLIT2 stimulation, and it also increases the activities of inflammatory signaling and cytoskeleton remodeling, which is a key step in cancer metastasis. In fact, the fundamental role of NEK9 was first noted in the regulation of mitosis, and it can phosphorylate key components for proper mitotic spindle formation [29]. Although the role of NEK9 in mitosis involves cytoskeletal reorganization, the effects of NEK9 on cancer cell progression and motility have rarely been investigated [30–33]. To the best of our knowledge, the authors research group was the first to systemically explore the roles of NEK9 in cancer metastasis [16]. It was found that NEK9 phosphorylated ARHGEF2, whose catalytic activity on RhoA activation was enhanced, leading to cytoskeletal reorganization [16]. In addition, NEK9 was also found to be an effector of IL-6/STAT3 through miR-520f-3p [16]. In the present study, NEK9 increased the total quantity and phosphorylation level of CTTN, which directly regulated cytoskeletal reorganization necessary for cancer cell movement. Notably, NEK9 can also enhance the transcription of STAT3 and NF-κB p100 by TRIM28. Combined with the findings from a series of studies [16], it was found that NEK9 was an effector of IL-6/STAT3, and also a key regulator of different inflammatory signaling pathways. In particular, IL-6 is also secreted by CAFs and confers metastasis-promoting effects [1, 2]. Therefore, pro-inflammatory cytokines and proteins derived from CAFs initiated a complex feedback loop between multiple inflammatory signaling pathways, creating a unique TME for GC metastasis. Recently, a multitargeted degrader technology using the ubiquitin-proteasome system was developed and targeting NEK9 using this new technology may provide novel therapeutic options for GC management [34].

Phosphorylation is a critical post-translational modification involved in the maintenance and enhancement of protein function, and this viewpoint was supported by the findings of the present study on the NEK9-mediated phosphorylation of TRIM28 and CTTN. TRIM28 was first identified as a novel STAT-binding partner [20]. Its transcriptional activities are attracting increasing attention in various types of cancers [35–37]. Phosphorylation is a determinant of its function, as its interaction with HIF-1 could lead to phosphorylation at serine-824 and activate target gene transcription in response to hypoxia [36]. Of note, serine-824 was also a phosphorylation site of NEK9 in the present study, although the phosphorylation on serine-824 did not exert any effects additional to NEK9 on GC metastasis. By contrast, phosphorylation on serine-473 markedly improved the metastasis-promoting effects of TRIM28. It appeared that not all phosphorylation sites were functional and the extent of phosphorylation of each functional site was highly variable. Therefore, the blockade, mutation or replacement of the functional phosphorylation serine residues of TRIM28 is another method, which can be used to control GC metastasis, and it may also avoid the side-effects of the non-selective silencing of NEK9. CTTN (coding gene of cortactin) contributes to the organization of the actin cytoskeleton and cell shape, and it also plays a role in the formation of lamellipodia and in cell migration [38, 39]. CTTN was the effector of the SLIT2/ROBO1/NEK9 pathway, and its expression and phosphorylation levels have a notable effect on GC cell morphology, which is consistent with its function in the cytoskeletal organization previously reported [38–40].

In the present study, a simultaneous increase in the expression levels of NEK9, TRIM28 and CTTN was found in metastatic GC lesions compared with paired non-cancerous tissues and primary cancer lesions. The analysis of the data of a cohort of patients with GC revealed that NEK9, TRIM28 and CTTN were associated with a reduced overall survival, and their levels were mutually positively associated. These findings were further validated by analysis from multiple online databases. The data of the clinical assessment suggested that the SLIT2/ROBO1/NEK9 pathway had clinical significance and provided preclinical support for the potential use of key molecules of this pathway as biomarkers and therapeutic targets for GC.

In conclusion, the present study found that CAF-derived SLIT2 promoted GC metastasis by enhancing the interaction between ROBO1 and NEK9. NEK9 functioned as a kinase by targeting phosphorylation of TRIM28 and CTTN, and triggered the activation of the IL-6/STAT3 and NF-κB pathways. NEK9 bridged the stimuli from CAFs and inflammatory signaling pathways, shaping a unique TME for cytoskeletal reorganization and cancer metastasis. These findings may contribute to an enhanced understanding of the role of the TME in GC metastasis.

## Supplementary information

Supplementary file

aj-checklist

Western blot raw data

## Data Availability

The data used and/or analyzed in the present study are available from the corresponding author on reasonable request.
