# Supplementary material for: Cancer associated fibroblast derived SLIT2 drives gastric cancer cell metastasis by activating NEK9
Source: Cell Death Dis. 2023 Jul 13;14(7):421. doi: 10.1038/s41419-023-05965-z (PMC10344862; doi:10.1038/s41419-023-05965-z)
Supplement: Supplementary file 1 — Supplementary file [file 41419_2023_5965_MOESM1_ESM.docx]

**Materials and methods**

**Patients and tissue microarrays.** The microarray used herein (T14-129, containing 105 gastric cancer cases; follow-up data were summarized at the end of October, 2020) with complete pathological and survival information was provided by the Xijing Hospital of Digestive Diseases, Fourth Military Medical University. The tissue microarrays, HStmA180Su15, HStmA180Su09, HStmAde180Sur02 and HStmAde076Met1, were purchased from Shanghai Outdo Biotech Co., Ltd.

**Luciferase reporter assay.** The Dual Luciferase Assay (Promega Corporation) was used to detect the luciferase activity according to standard instructions. Using a microcentrifuge, the transfected cells were lysed in a lysis buffer and centrifuged for 1 min at 12000 x g. A Modulus TM TD20/20 Luminometer (Turner Biosystems) was used to measure the relative luciferase activity. Transfection efficiencies were normalized using *Renilla* activity.

**Immunoprecipitation (IP).** Protein A/G Magnetic Beads (IC-8110, InCellGene), and the primary antibodies anti-IgG (cat. no. B900610, ProteinTech Group, Inc.), anti-TRIM28 (cat. no. 15202-1-AP, ProteinTech Group, Inc.), anti- Flag (cat. no. MA5558, AntiProtech Inc.), anti-ROBO1(cat. no. 20219-1-AP, ProteinTech Group, Inc.), anti-CTTN (cat. no. sc-55579, Santa Cruz Biotechnology, Inc.) and anti-NEK9 (cat. no. sc-100401, Santa Cruz Biotechnology, Inc.) were used for the IP assay according to the manufacturer's instructions. The samples were detected using western blot analysis.

**Chromatin immunoprecipitation assay (ChIP).** The cells that were transfected with the appropriate plasmids were cross-linked in 1% formaldehyde at 37˚C for 10 min. The resuspended cells were resuspended in lysis buffer (50 mM Tris, pH 8.1, 10 mM EDTA, 1% SDS and 1 mM PMSF) after washing with PBS. Sonication was used to break the DNA into small fragments, and G-Sepharose slurry (Millipore Sigma-Aldrich) was used to pre-clear the supernatants. The recovered supernatants were incubated with specific antibodies or an isotype control IgG for 2 h in the presence of herring sperm DNA and protein G-Sepharose beads. The PCR Purification kit (Qiagen, Inc.) was used to purify immunoprecipitated DNA from the beads using 1% SDS and 1.1 M NaHCO_3_. The primers used are presented in Tables S2-S4.

**Analysis using MS.** Proteins were extracted from the MKN45 and AGS cells stably overexpressing NEK9, from cells in which NEK9 was knocked down NEK9 and the controls. A total of 150 μg protein from each sample was digested with different enzymes, and the digested peptide (100 μg) from the different samples was labeled using tandem mass tags (TMT) reagents (Pierce; Thermo Fisher Scientific, Inc.). Phosphopeptide enrichment was performed on 5 µm titansphere bulk particles (Canadian Life Science) according to manufacturer's protocols.

GC cell lysates were collected and preclear with 20ul (bed volume) protein A sepharose for 1 hour at 4^o^C, and added anti-ROBO1 antibody-conjugated agarose and incubate for 4 hours to O/N at 4^o^C. Then, washed 3 x TRITON100 lysis buffer then 2 x 20 mM ABC. Add 0.4ug Trypsin (2ul from 200ng/ul stock), incubate at 37^o^C from 6 hours to O/N. Refresh trypsin once if do O/N digestion. After digestion, add 100ul 3% formic acid into each tube. Spin at 14,000rpm x 3min. Transfer liquid into new low-retention tubes. The data were analyzed using nano-LC-MS/MS. comprehensive data mining and filtering identified differentially expressed genes in different proteins form each sample.

**Supplementary figures**


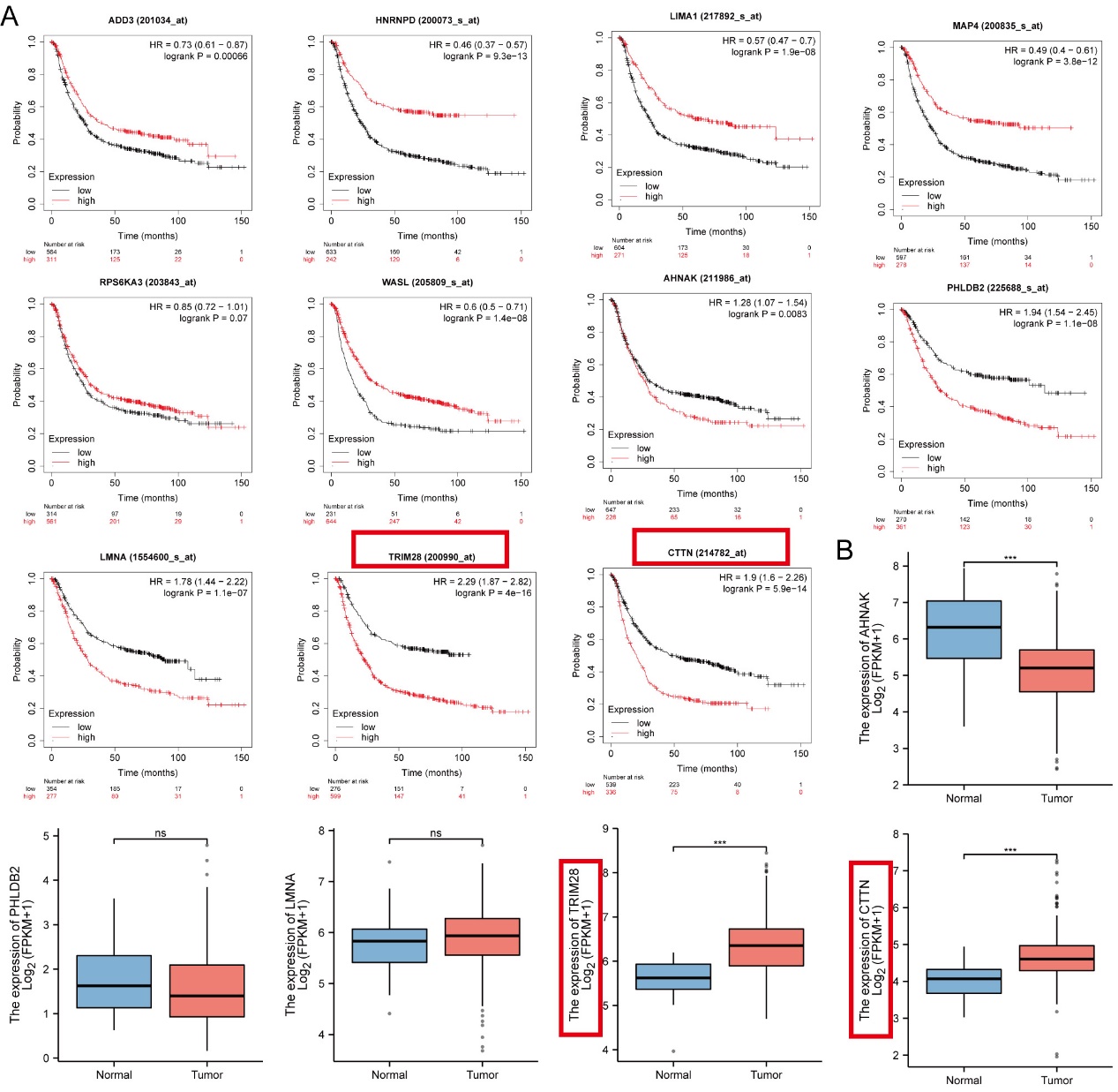


Figure S1. TRIM28 and CTTN expression levels were increased in GC and associated with a decreased survival rate. (A) Survival analysis using the Kaplan-Meier plotter tool in ADD3, HNRNPD, LIMA1, MAP4, RPS6KA3, WASL, AHNAK, PHLDB2, LMNA, TRIM28 and CTTN of overall survival. (B) The expression of AHNAK, PHLDB2, LMNA, TRIM28 and CTTN were analyzed from The Cancer Genome Atlas (TCGA).


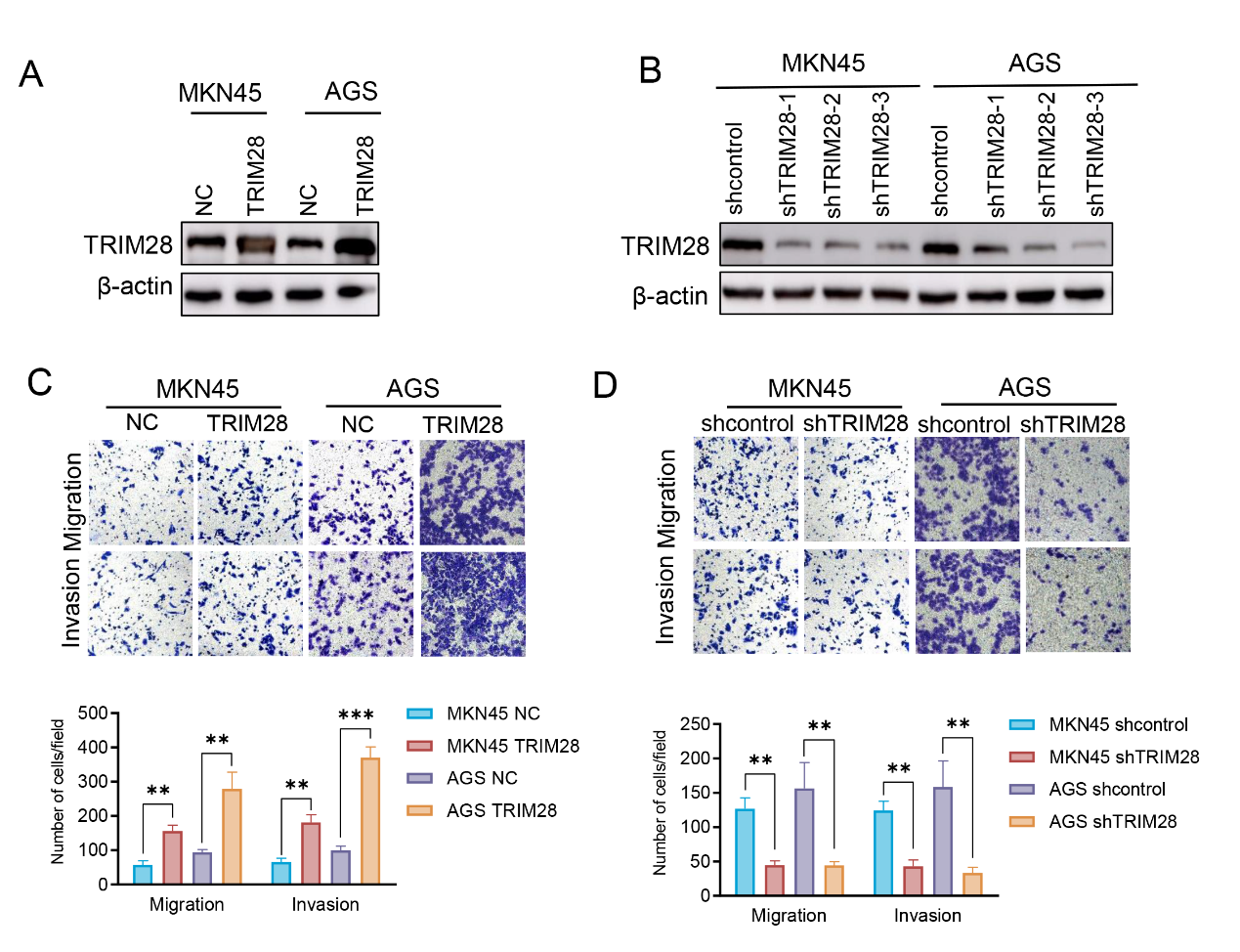


Figure S2. TRIM28 promoted Gastric cancer cells metastasis *in vitro*. Cell models with stable Levi - TRIM28 overexpression(A) and shTRIM28 plasmids (B) transfection were established and validated by western blotting. (C-D) TRIM28 promoted cell migration and invasion (C), while its knockdown inhibited cell movement (D) via *in vitro* transwell analysis. ***P* < 0.01, ****P* < 0.001.


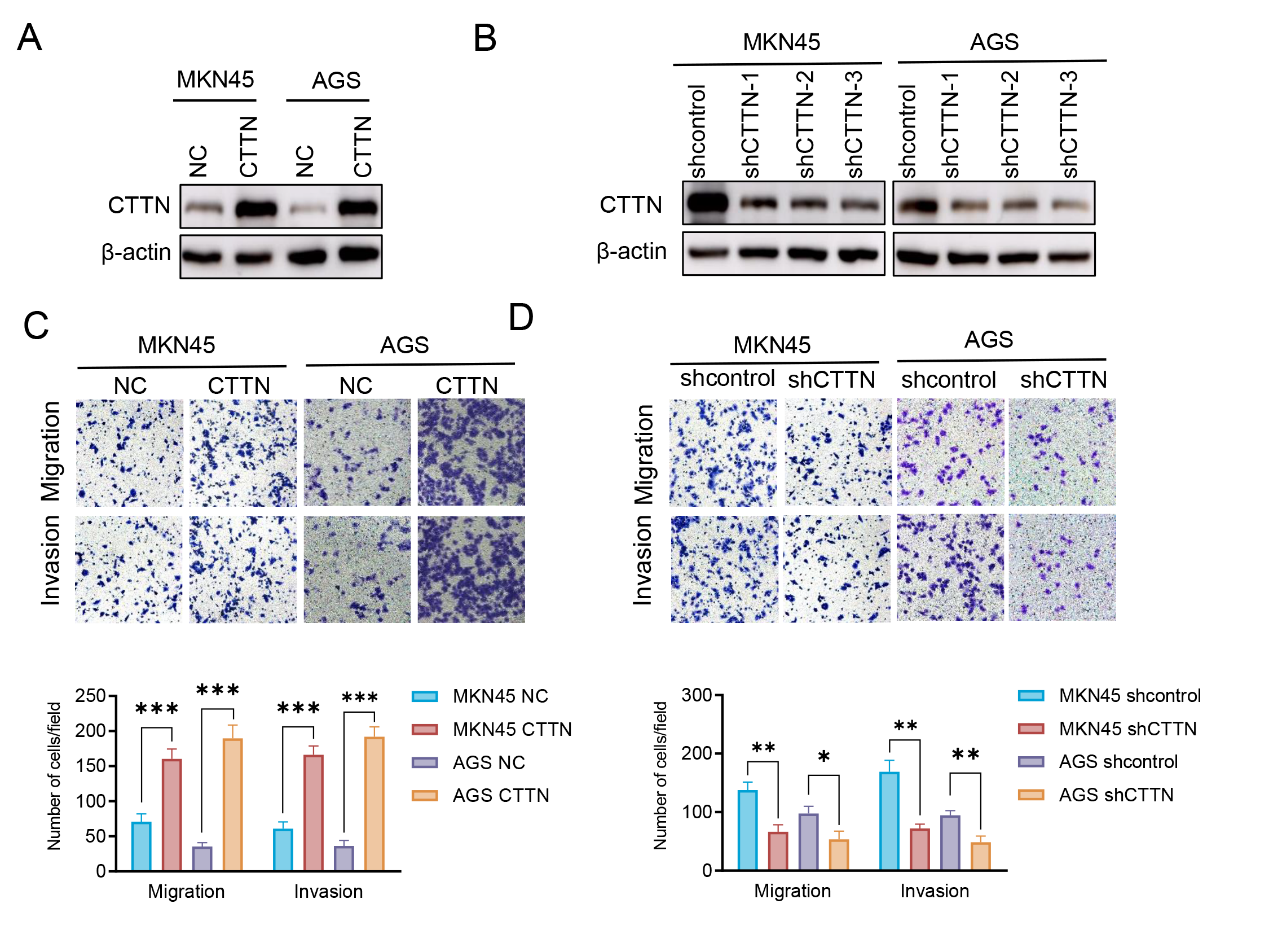


Figure S3. CTTN promoted Gastric cancer cells metastasis *in vitro*. Cell models with stable Levi – CTTN overexpression(A) and shCTTN plasmids (C) transfection were established and validated by western blotting. (B, D) CTTN promoted cell migration and invasion (B), while its knockdown inhibited cell movement (D) via *in vitro* transwell analysis. **P* < 0.05, ***p* < 0.01, ****P* < 0.001.


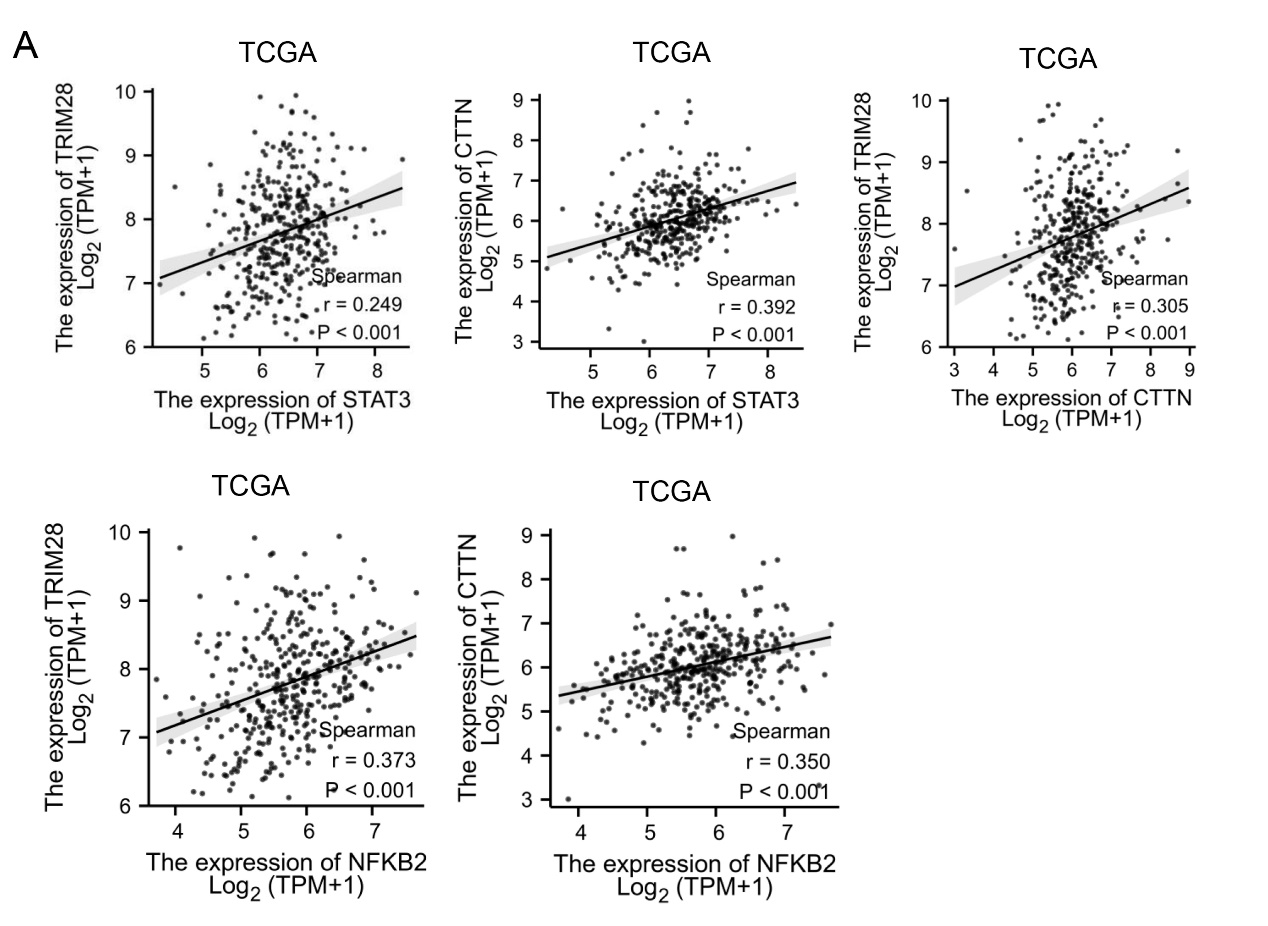


Figure S4. TCGA data analysis were used to reveal the positive association between TRIM28, STAT3 and NFκB2 p100. (A) The association between TRIM28, STAT3 and NFκB2 p100 were analyzed from TCGA.


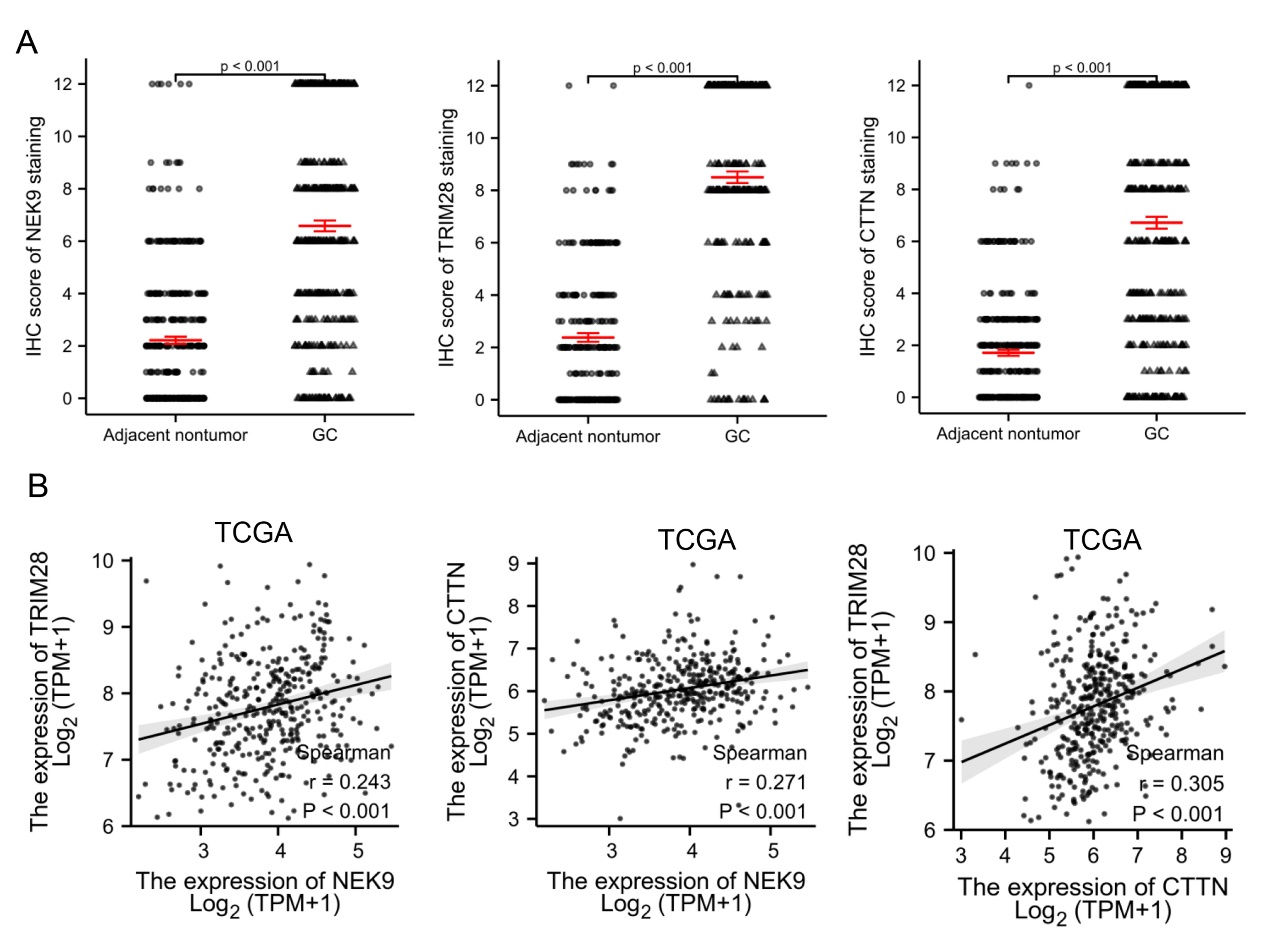


Figure S5. NEK9, TRIM28 and CTTN expression levels were increased in GC and positive association between each other. (A) The NEK9, TRIM28 and CTTN expression in Adjacent nontumor and Gastric cancer tissues were analyzed from TCGA. (B) The association between NEK9, TRIM28 and CTTN were analyzed from TCGA.

**Supplementary Tables**

Table S1. List of increased and decreased phosphorylation genes differentially expression after NEK9 overexpression and knockdown.

| Gene name | Gene description |
| --- | --- |
| ADD3 | Gamma-adducin |
| HNRNPD | Heterogeneous nuclear ribonucleoprotein |
| LIMA1 | LIM domain and actin-binding protein 1 |
| MAP4 | Mitogen-activated protein kinase 4 |
| RPS6KA3 | Ribosomal protein S6 kinase alpha-3 |
| WASL | Neural Wiskott-Aldrich syndrome protein |
| AHNAK | Neuroblast differentiation-associated protein |
| PHLDB2 | Pleckstrin homology-like domain family B member 2 |
| LMNA | Prelamin-A/C |
| TRIM28 | Transcription intermediary factor 1-beta |
| CTTN | Src substrate cortactin |
| SRRM2 | Serine/arginine repetitive matrix protein 2 |
| CAD | CAD protein |
| SRSF2 | Serine/arginine-rich splicing factor 2 |
| CARHSP1 | Calcium-regulated heat-stable protein 1 |
| TPD52L2 | Tumor protein D54 |
| STK24 | Serine/threonine-protein kinase 24 |
| MAP1S | Microtubule-associated protein 1S |
| SAMHD1 | Deoxynucleoside triphosphate triphosphohydrolase |
| NUCKS1 | Nuclear ubiquitous casein and cyclin-dependent kinase substrate 1 |
| IMUP | Immortalization up-regulated protein |
| CCNL1 | Cyclin-L1 |
| RPL31 | 60S ribosomal protein L31 |
| PGK1 | Phosphoglycerate kinase 1 |
| SRRM1 | Serine/arginine repetitive matrix protein 1 |
| EIF3D | Eukaryotic translation initiation factor 3 subunit D |
| SPEN | Msx2-interacting protein |
| EPRS | Bifunctional glutamate/proline--tRNA ligase |
| HDGF | Hepatoma-derived growth factor |
| MTSS1L | MTSS1-like protein |
| MKI67 | Proliferation marker protein Ki-67 |
| PPIG | Peptidyl-prolyl cis-trans isomerase G |
| OTUD7B | OTU domain-containing protein 7B |
| LARP1 | La-related protein 1B |
| CDK12 | Cyclin-dependent kinase 12 |
| STX4 | Syntaxin-4 |
| FAM208A | Protein TASOR |
| ARHGEF2 | Rho guanine nucleotide exchange factor 2 |
| PARP4 | Poly [ADP-ribose] polymerase 4 |
| ANKRD11 | Ankyrin repeat domain-containing protein 11 |
| TMOD3 | Tropomodulin-3 |
| ARHGAP35 | Rho GTPase-activating protein 35 |
| NES | Nestin |
| JUND | Transcription factor jun-D |
| CLASP2 | CLIP-associating protein 2 |
| CDK11B | Cyclin-dependent kinase 11B |
| SRSF1 | Serine/arginine-rich splicing factor 1 |
| NOP14 | Nucleolar protein 14 |
| MTA1 | Metastasis-associated protein MTA1 |
| CBX5 | Chromobox protein homolog 5 |
| DSP | Desmoplakin |
| SLC12A4 | Solute carrier family 12 member 4 |
| PELP1 | Proline-, glutamic acid- and leucine-rich protein 1 |
| FAM83H | Protein FAM83H |
| DDX10 | Probable ATP-dependent RNA helicase DDX10 |
| FOSL2 | Fos-related antigen 2 |
| AHCTF1 | Protein ELYS |

Table S2. Primer sequences used for TRIM28 binding site on CTTN promoter.

| Primer name | Primer sequences |
| --- | --- |
| Primers used for Ch-IP in the CTTN promoter | |
| CTTN Ch-IP NC sense: | 5'TTAGGGTGGGCTAAACATCTG 3' |
| CTTN Ch-IP NC antisense: | 5'CTCCTCCTCTGCCTGACCTG 3' |
| CTTN Ch-IP 1 sense: | 5'GCAGAGGTTGCAGTGAGCTG 3' |
| CTTN Ch-IP 1 antisense: | 5'GTTTGTTTAATCTTTAGAGACAG 3' |
| CTTN Ch-IP 2 sense: | 5'GCCCAGGCTGGTCTTGAACTC 3' |
| CTTN Ch-IP 2 antisense: | 5'GCCCAGGCTGGTCTTGAACTC 3' |
| CTTN Ch-IP 3 sense: | 5'CAGCTCCGCTGCACCACTAG 3' |
| CTTN Ch-IP 3 antisense: | 5'AAGAATTTTCCAGGCAGGCC 3' |
| CTTN Ch-IP 4 sense: | 5'TGAGCGGACGAGGACGGCTG 3' |
| CTTN Ch-IP 4 antisense: | 5'GAGTCTGCAGAGCTCGCCCG 3' |
| Primers for CTTN promoter construct: | |
| CTTN-1 sense KpnI: | 5'cggggtaccAGGCAGAGGTTGCAGTGAGCTGAGATGGCG 3' |
| CTTN-2 sense KpnI: | 5'cggggtaccGTCTCTAAAGATTAAACAAACAAACAA 3' |
| CTTN-3 sense KpnI: | 5'cggggtaccGCAAATTAGCTGTGTCCAGTTAAATGCA 3' |
| CTTN-4 sense KpnI: | 5'cggggtaccAGGTCTTCGCTGTGCTCTATCGGCCTCT 3' |
| CTTN-5 sense KpnI: | 5'cggggtaccCCTGCTGTTGGCCTGCCTGGAAAATTCT 3' |
| CTTN-6 sense KpnI: | 5'cggggtaccTGTGTGCTGCTCAGCGCCCATCCTCTGC 3' |
| Antisense HindIII: | 5'ccgctcgagCCCAGGTTGGAAGGGGTAGCAGGCATGTCG 3' |

Table S3. Primer sequences used for STAT3 binding site on CTTN promoter.

| Primer name | Primer sequences |
| --- | --- |
| Primers used for Ch-IP in the CTTN promoter | |
| CTTN Ch-IP NC sense: | 5'ATCATGGTTCATTGGCAGCC 3' |
| CTTN Ch-IP NC antisense: | 5'CAGGCACAGTGACTCATGCC 3' |
| CTTN Ch-IP 1 sense: | 5'CTGTTGCCCGTGCCGGAGCG 3' |
| CTTN Ch-IP 1 antisense: | 5'AGAATTTGGCCAGATGCAGTG 3' |
| CTTN Ch-IP 2 sense: | 5'GCCCAAACTTCATGATGTCTC 3' |
| CTTN Ch-IP 2 antisense: | 5'AAGAATTTTCCAGGCAGGCC 3' |
| CTTN Ch-IP 3 sense: | 5'CGTCTCCCCAATACGATGTTATG 3' |
| CTTN Ch-IP 3 antisense: | 5'CTCCCCTCCAAGAAAGGGCCAC 3' |
| Primers for CTTN promoter construct: | |
| CTTN-1 sense KpnI: | 5'cggggtaccGCAAATTAGCTGTGTCCAGTTAAAT 3' |
| CTTN-2 sense KpnI: | 5'cggggtaccAAATTTTTTTTTTTTGGTAGAGACAGGGTC 3' |
| CTTN-3 sense KpnI: | 5'cggggtaccAAAGACCGAGGTCTTCGCTGTGCTCTATCG 3' |
| CTTN-4 sense KpnI: | 5'cggggtaccATTCTTCCCTCCTTACCTTGTTTACTCTTA 3' |
| CTTN-5 sense KpnI: | 5'cggggtaccGGAGGTGCATGGTGAGCGGACGAGGACGGC 3' |
| CTTN-6 sense KpnI: | 5'cggggtaccCTGGGTTCCGGCTCACACTTCTGCC 3' |
| Antisense HindIII: | 5'ccgctcgagCCCAGGTTGGAAGGGGTAGCAGGCATGTCG 3' |

Table S4. Primer sequences used for p100 binding site on CTTN promoter.

| Primer name | Primer sequences |
| --- | --- |
| Primers used for Ch-IP in the CTTN promoter | |
| CTTN Ch-IP NC sense: | 5'TTCTCCTGCCTCAGCCTCCC 3' |
| CTTN Ch-IP NC antisense: | 5'GGAGGACGAGGTGGGAGGATAAC 3' |
| CTTN Ch-IP 1 sense: | 5'AGGCAGAGGTTGCAGTGAGC 3' |
| CTTN Ch-IP 1 antisense: | 5'TGTTTGTTTAATCTTTAGAGAC 3' |
| CTTN Ch-IP 2 sense: | 5'ACCTCTGGTCTCCCAGGGGTTC 3' |
| CTTN Ch-IP 2 antisense: | 5'GCCAGGGACGCGAGCCCCGGC 3' |
| Primers for CTTN promoter construct: | |
| CTTN-1 sense KpnI: | 5'cggggtaccAGGCAGAGGTTGCAGTGAGCTGAGATGGCG 3' |
| CTTN-2 sense KpnI: | 5'cggggtaccCTCAAAAAAAAAAAAATTAGCTGAGTGTGG 3' |
| CTTN-3 sense KpnI: | 5'cggggtaccTGTGGTTTAACGCTTGACTCACCTTCTAGGC 3' |
| Antisense HindIII: | 5'ccgctcgagCCCAGGTTGGAAGGGGTAGCAGGCATGTCG 3' |
|  | |
|  |  |

Table S5. The relationship between NEK9 expression and clinicopathological characteristics in GC

| Characteristic | Low | High | p |
| --- | --- | --- | --- |
| n | 88 | 108 |  |
| sex, n (%) |  |  | 0.620 |
| female | 28 (31.8) | 38 (35.2) |  |
| male | 60 (68.2) | 70 (64.8) |  |
| Pathologic stage, n (%) |  |  | 0.032 |
| Ⅰ+Ⅱ | 40 (45.5) | 33 (30.6) |  |
| Ⅲ+Ⅳ | 48 (54.5) | 75 (69.4) |  |
| T, n (%) |  |  | 0.032 |
| T1 | 8 (9.1) | 8 (7.4) |  |
| T2 | 16 (18.2) | 17 (15.7) |  |
| T3 | 60 (68.2) | 63 (58.3) |  |
| T4 | 4 (4.5) | 20 (18.5) |  |
| N, n (%) |  |  | 0.281 |
| N0+N1 | 34 (38.6) | 50 (46.3) |  |
| N2+N3 | 54 (61.4) | 58 (53.7) |  |
| M, n (%) |  |  | 0.046 |
| M0 | 78 (88.6) | 84 (77.8) |  |
| M1 | 10 (11.4) | 24 (22.2) |  |
| Clinical stage, n (%) |  |  | 0.039 |
| 1 | 13 (14.8) | 9 (8.3) |  |
| 2 | 36 (40.9) | 34 (31.5) |  |
| 3 | 35 (39.8) | 49 (45.4) |  |
| 4 | 4 (4.5) | 16 (14.8) |  |

Table S6. The relationship between TRIM28 expression and clinicopathological characteristics in GC

| Characteristic | Low | High | p |
| --- | --- | --- | --- |
| n | 96 | 100 |  |
| sex, n (%) |  |  | 0.752 |
| female | 42 (43.8) | 46 (46.0) |  |
| male | 54 (56.3) | 54 (54.0) |  |
| Pathologic stage, n (%) |  |  | 0.497 |
| Ⅰ+Ⅱ | 43 (44.8) | 40 (40.0) |  |
| Ⅲ+Ⅳ | 53 (55.2) | 60 (60.0) |  |
| T, n (%) |  |  | 0.054 |
| T1 | 19 (19.8) | 9 (9.0) |  |
| T2 | 21 (21.9) | 15 (15.0) |  |
| T3 | 40 (41.7) | 52 (52.0) |  |
| T4 | 16 (16.7) | 24 (24.0) |  |
| N, n (%) |  |  | 0.160 |
| N0+N1 | 47 (49.0) | 39 (39.0) |  |
| N2+N3 | 49 (51.0) | 61 (61.0) |  |
| M, n (%) |  |  | 0.033 |
| M0 | 85 (88.5) | 77 (77.0) |  |
| M1 | 11 (11.5) | 23 (23.0) |  |
| Clinical stage, n (%) |  |  | 0.038 |
| 1 | 24 (25.0) | 16 (16.0) |  |
| 2 | 35 (36.5) | 30 (30.0) |  |
| 3 | 30 (31.3) | 34 (34.0) |  |
| 4 | 7 (7.3) | 20 (20.0) |  |

Table S7. The relationship between CTTN expression and clinicopathological characteristics in GC

| Characteristic | Low | High | p |
| --- | --- | --- | --- |
| n | 79 | 117 |  |
| sex, n (%) |  |  | 0.356 |
| female | 24 (30.4) | 43 (36.8) |  |
| male | 55 (69.6) | 74 (63.2) |  |
| Pathologic stage, n (%) |  |  | 0.642 |
| Ⅰ+Ⅱ | 33 (41.8) | 45 (38.5) |  |
| Ⅲ+Ⅳ | 46 (58.2) | 72 (61.5) |  |
| T, n (%) |  |  | 0.049 |
| T1 | 11 (13.9) | 9 (7.7) |  |
| T2 | 20 (25.3) | 21 (17.9) |  |
| T3 | 45 (57.0) | 71 (60.7) |  |
| T4 | 3 (3.8) | 16 (13.7) |  |
| N, n (%) |  |  | 0.043 |
| N0+N1 | 37 (46.8) | 38 (32.5) |  |
| N2+N3 | 42 (53.2) | 79 (67.5) |  |
| M, n (%) |  |  | 0.010 |
| M0 | 72 (91.1) | 90 (76.9) |  |
| M1 | 7 (8.9) | 27 (23.1) |  |
| Clinical stage, n (%) |  |  | 0.014 |
| 1 | 16 (20.3) | 8 (6.8) |  |
| 2 | 29 (36.7) | 45 (38.5) |  |
| 3 | 33 (41.8) | 56 (47.9) |  |
| 4 | 1 (1.3) | 8 (6.8) |  |
