# Supplementary material for: Cancer associated fibroblast derived SLIT2 drives gastric cancer cell metastasis by activating NEK9
Source: Cell Death Dis. 2023 Jul 13;14(7):421. doi: 10.1038/s41419-023-05965-z (PMC10344862; doi:10.1038/s41419-023-05965-z)
Supplement: Supplementary file 3 — Western blot raw data [file 41419_2023_5965_MOESM3_ESM.pdf]

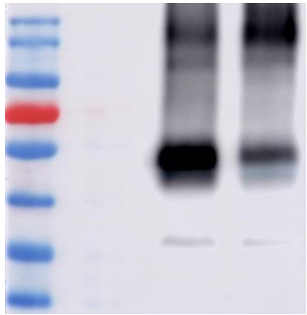

figure 2C
IP ROBO1-NEK9

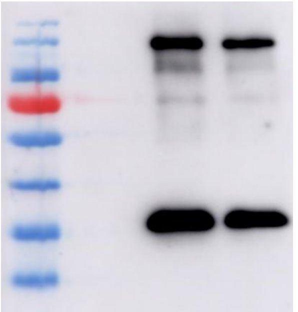

figure 2C
IP-ROBO1-ROBO1

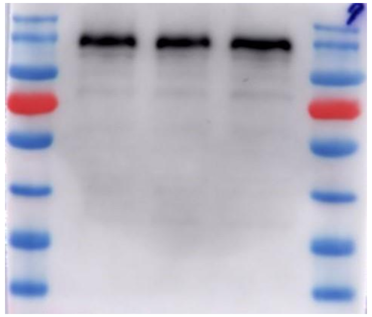

figure 2C
WCL-NEK9

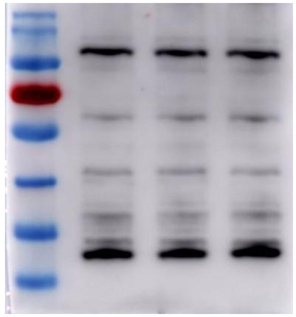

figure 2C
WCL-ROBO1

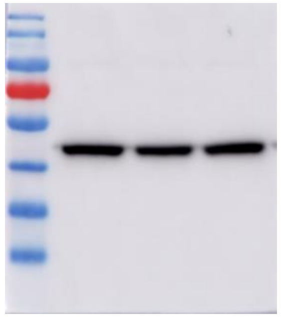

figure 2C
WCL-β-actin

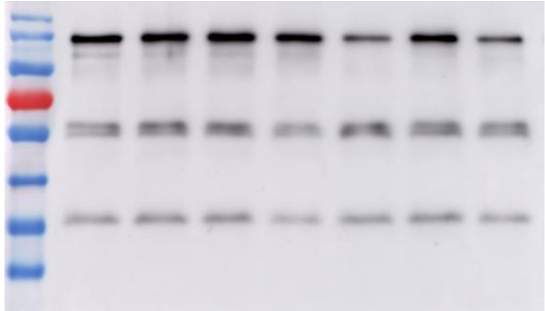
figure 2D
IP-Flag-Flag

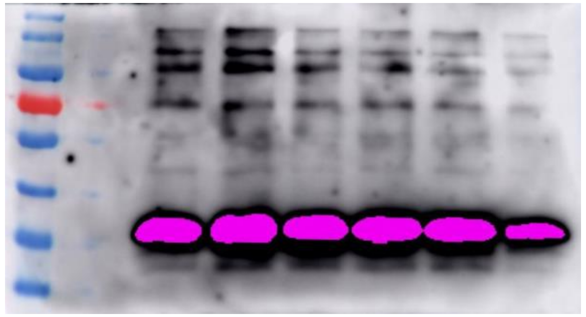
figure 2D
IP-Flag-HA

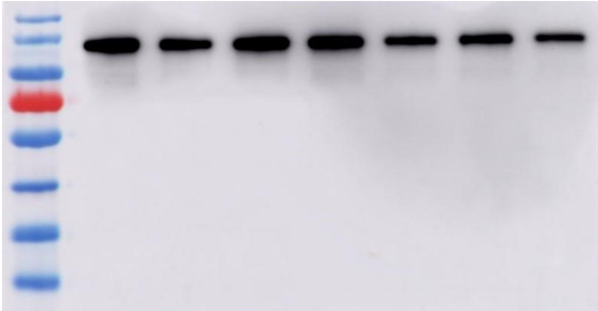
figure 2D
WCL-Flag

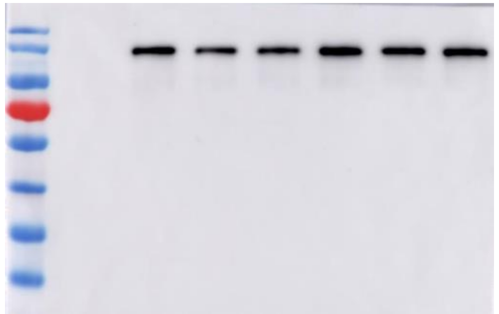
figure 2D
WCL-HA

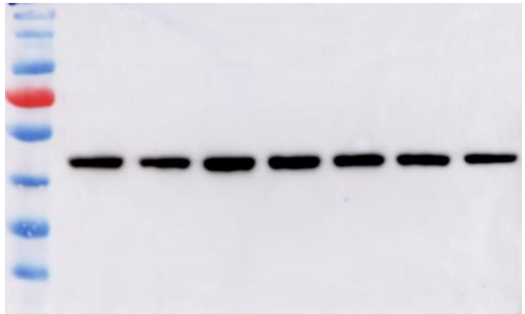
figure 2D
WCL-βactin

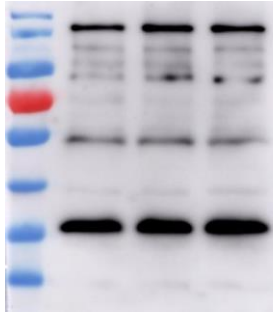

figure 2E
IP-Flag

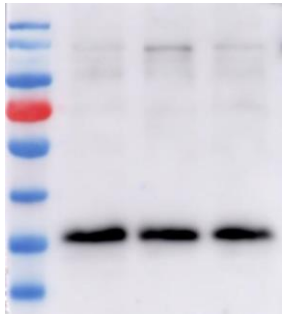

figure 2E
IP-HA

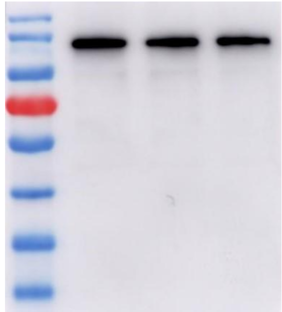

figure 2E
WCL-Flag

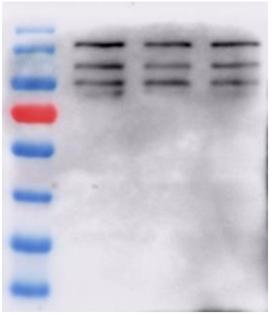

figure 2E
WCL-HA

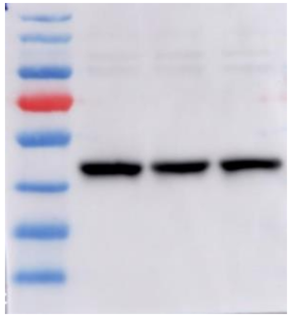

figure 2E
WCL-β-actin

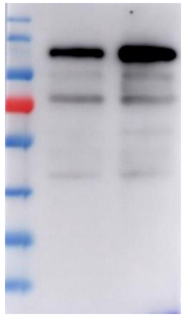
figure 3D
AGS IP p-ser

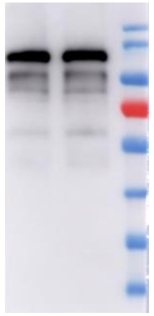
figure 3D
AGS IP TRIM28

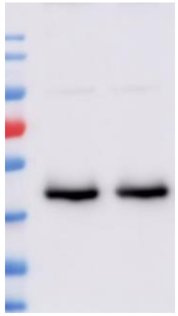
figure 3D
AGS WCL actin

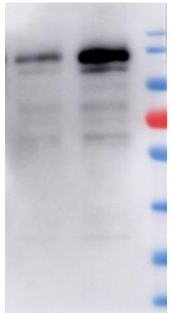
figure 3D
AGS WCL NEK9

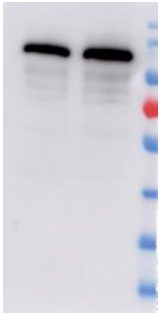
figure 3D
AGS WCL TRIM28

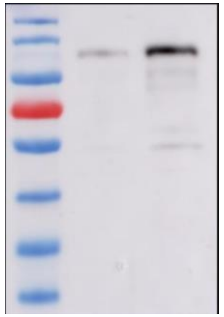
figure 3D
MKN45 IP p-ser

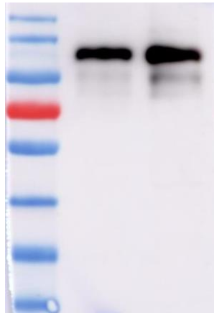
figure 3D
MKN45 IP TRIM28

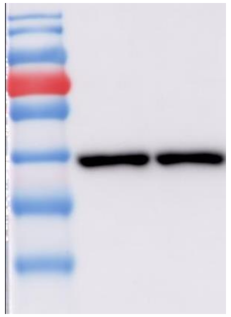
figure 3D
MKN45 WCL actin

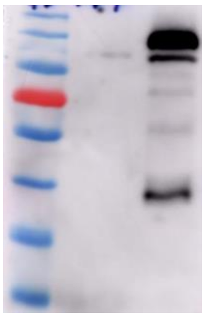
figure 3D
MKN45 WCL NEK9

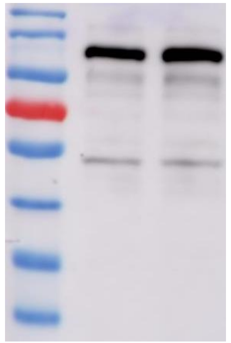
figure 3D
MKN45 WCL TRIM28

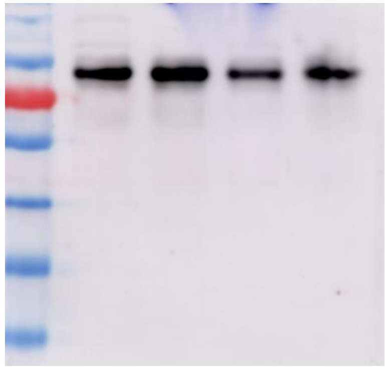

figure 3E
CTTN

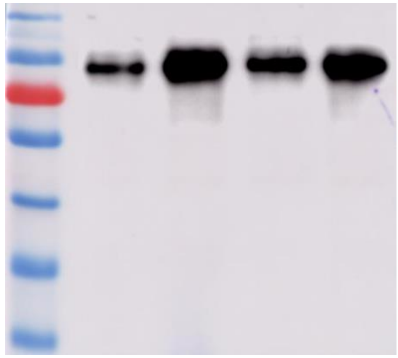

figure 3E
NEK9

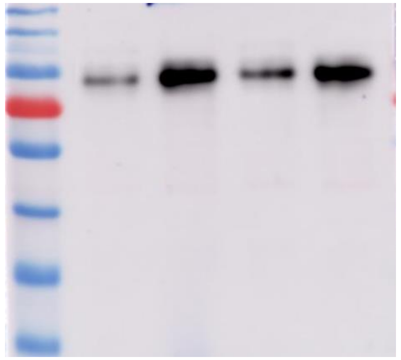

figure 3E
p-CTTN

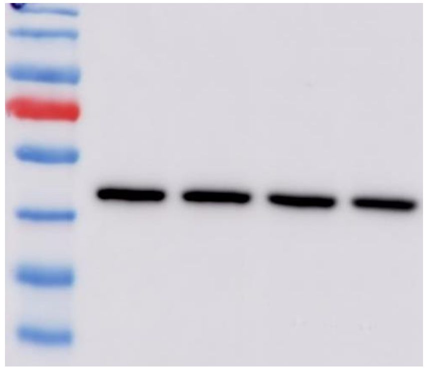

figure 3E
β-actin

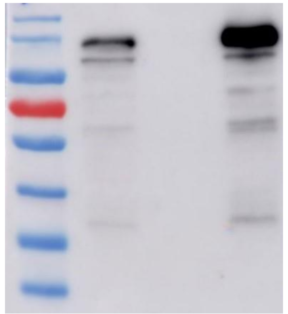

figure 3G
AGS NEK9-NEK9

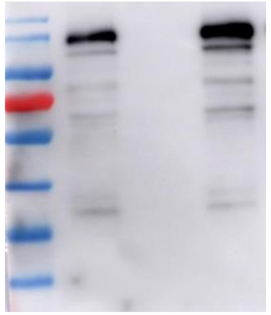

figure 3G
AGS NEK9-TRIM28

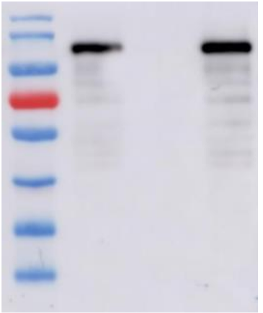

figure 3G
AGS TRIM28-NEK9

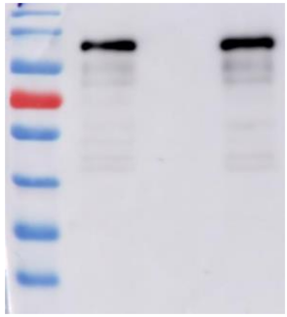

figure 3G
AGS TRIM28-TRIM28

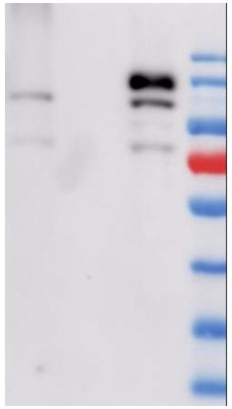

figure 3G
MKN45 NEK9-NEK9

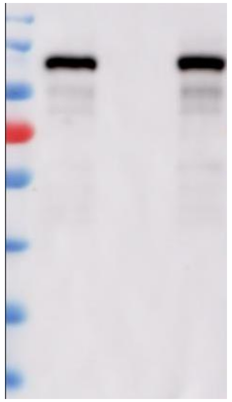

figure 3G
MKN45 NEK9-TRIM28

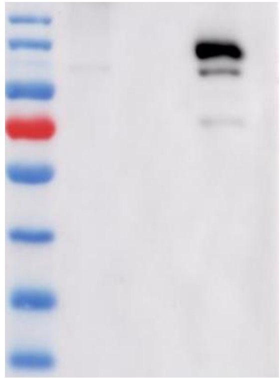

figure 3G
MKN45 TRIM28-NEK9

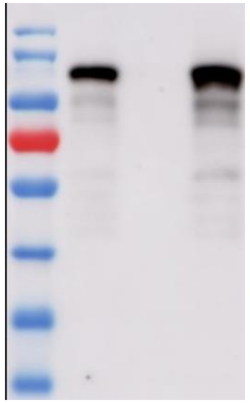

figure 3G
MKN45 TRIM28-TRIM28

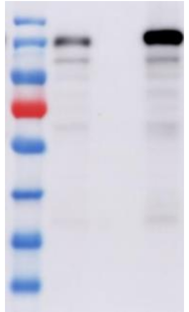

figure 3H
AGS CTTN-CTTN

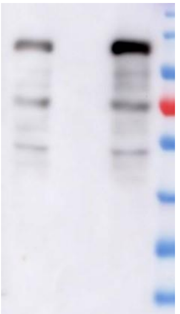

figure 3H
AGS CTTN-NEK9

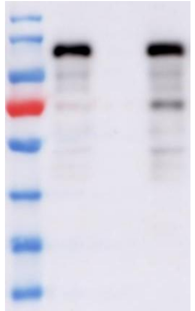

figure 3H
AGS NEK9-CTTN

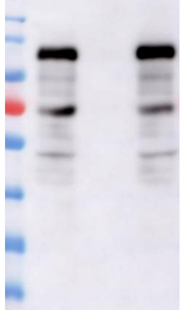

figure 3H
AGS NEK9-NEK9

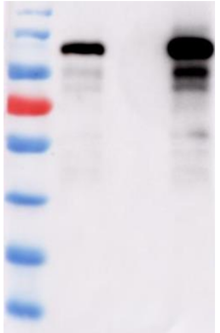

figure 3H
MKN45 NEK9-CTTN

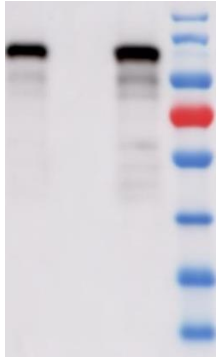

figure 3H
MKN45 NEK9-NEK9

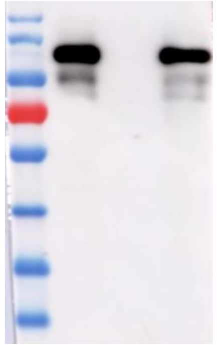

figure 3H
MKN45 CTTN-CTTN

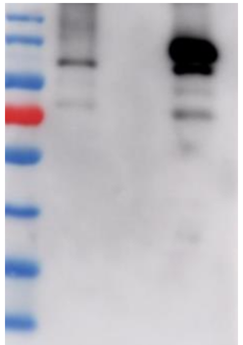

figure 3H
MKN45 CTTN-NEK9

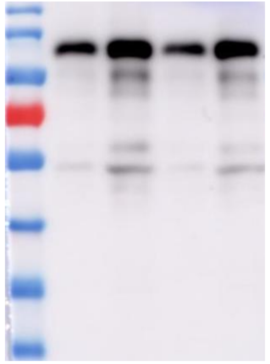

figure 3L
p-Ser

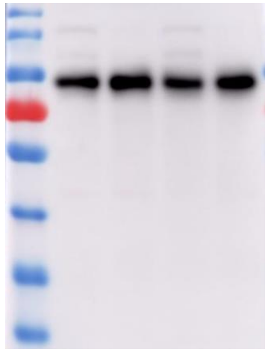

figure 3L
p-CTTN

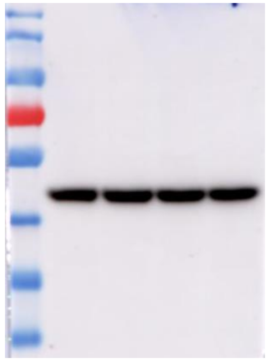

figure 3L
β-actin

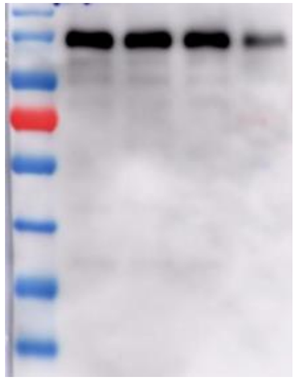

figure 4A
IP-Flag

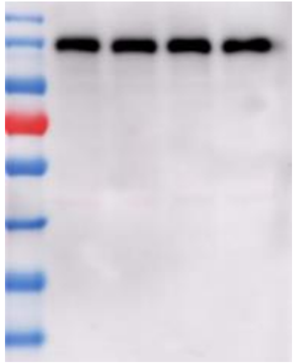

figure 4A
WCL-Flag

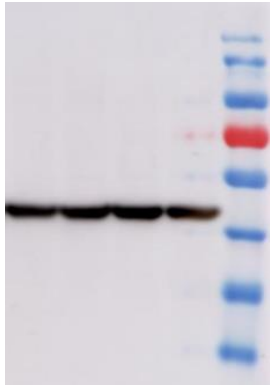

figure 4A
WCL-β-actin

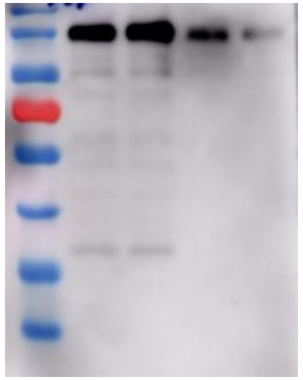

figure 4A
IP-Ser

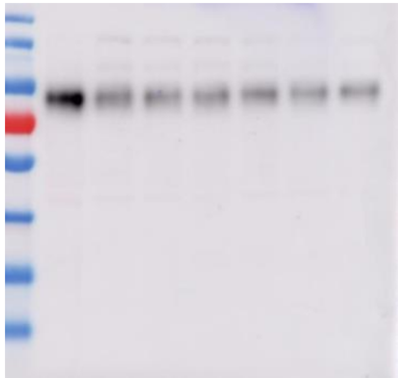

figure 4B1
IP-Flag

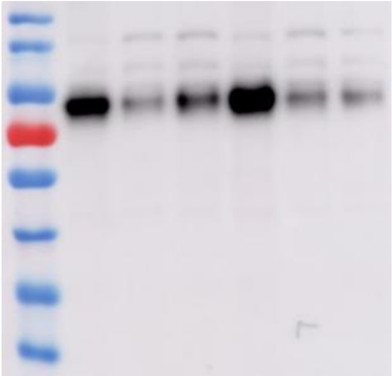

figure 4B1
IP-Ser

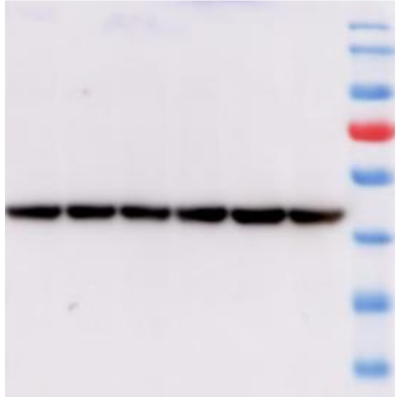

figure 4B1
WCL-Flag

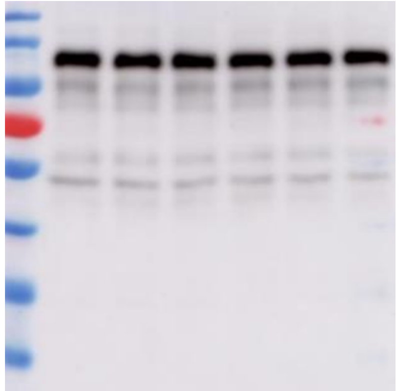

figure 4B1
WCL-β-actin

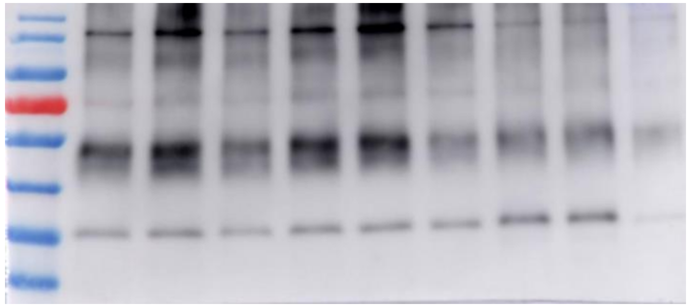

figure 4C1
IP-Flag

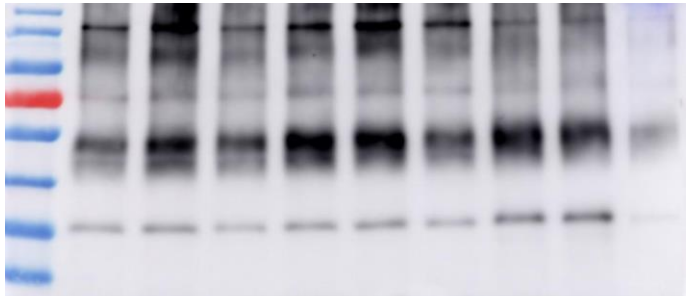

figure 4C1
IP-Ser

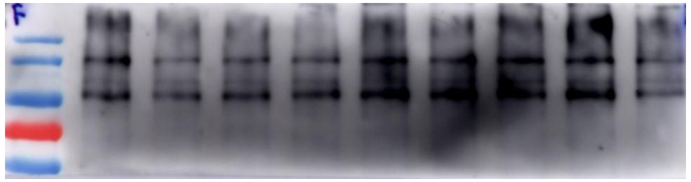

figure 4C1
WCL-Flag

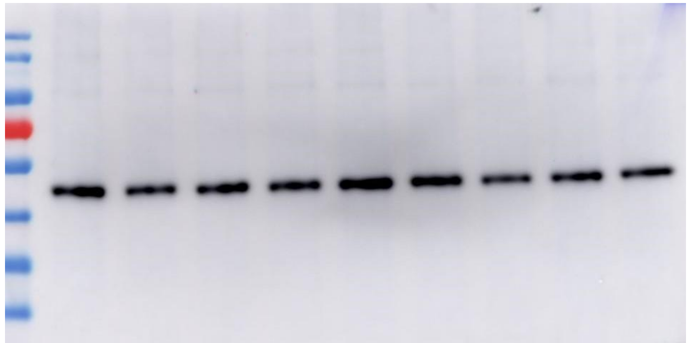

figure 4C1
WCL-β-actin

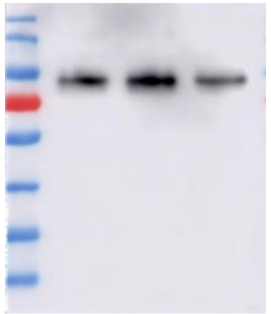

figure 4D1
AGS-CTTN

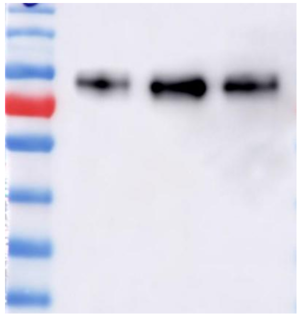

figure 4D1
AGS-pCTTN

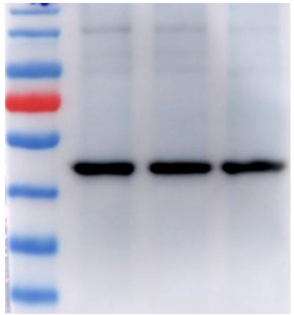

figure 4D1
AGS-β-actin

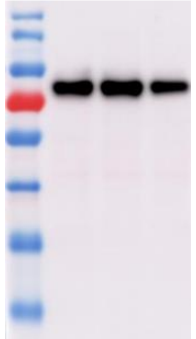

figure 4D1
MKN45-CTTN

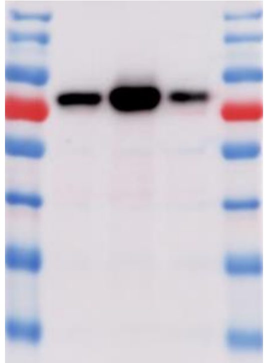

figure 4D1
MKN45-pCTTN

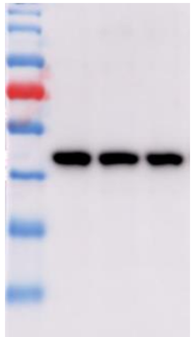

figure 4D1
MKN45-β-actin

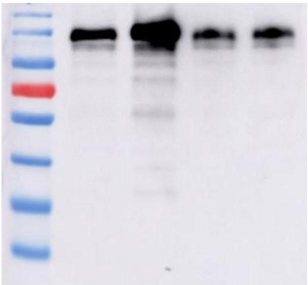

figure 5A
AGS CTTN

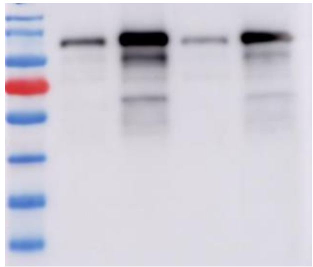

figure 5A
AGS TRIM28

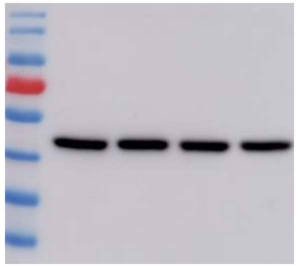

figure 5A
AGS β-actin

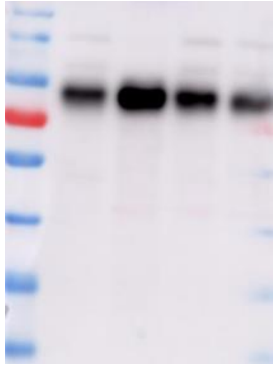

figure 5A
MKN45 CTTN

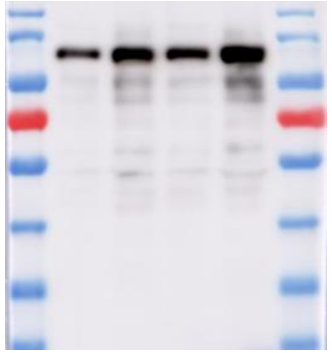

figure 5A
MKN45 TRIM28

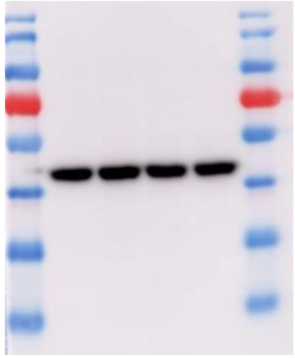

figure 5A
MKN45 β-actin

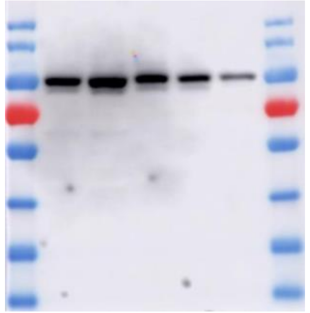

figure 6A
AGS-p100

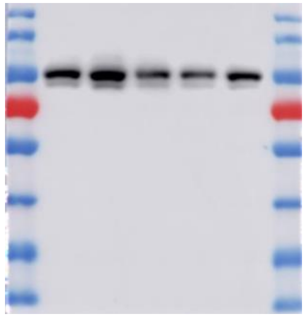

figure 6A
AGS-pSTAT3

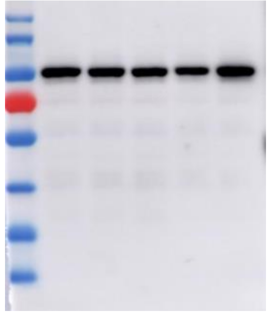

figure 6A
AGS-STAT3

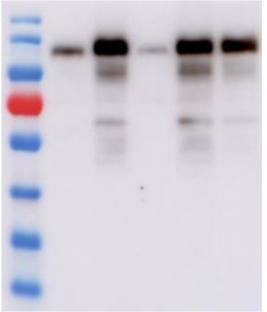

figure 6A
AGS-TRIM28

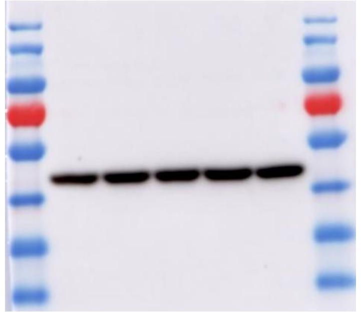

figure 6A
AGS-β-actin

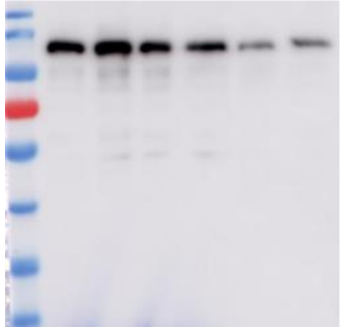

figure 6A
MKN45-p100

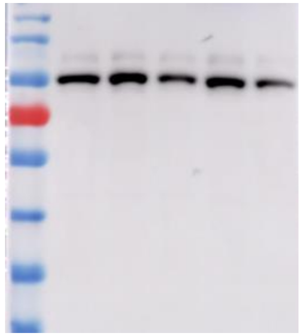

figure 6A
MKN45-pSTAT3

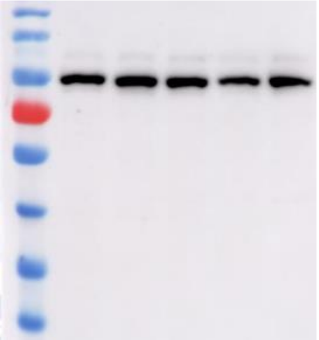

figure 6A
MKN45-STAT3

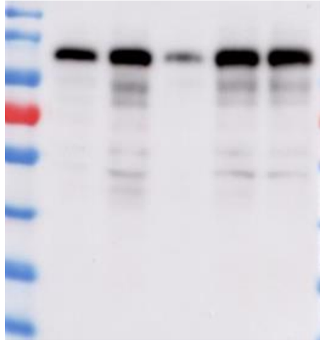

figure 6A
MKN45-TRIM28

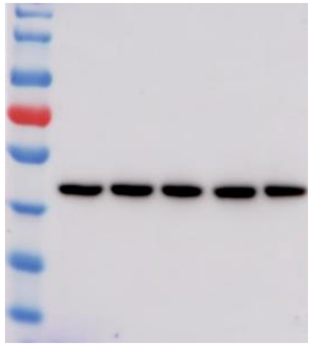

figure 6A
MKN45-β-actin

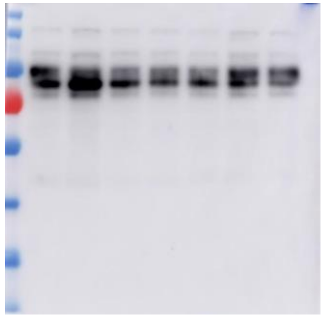

figure 6C
AGS-CTTN

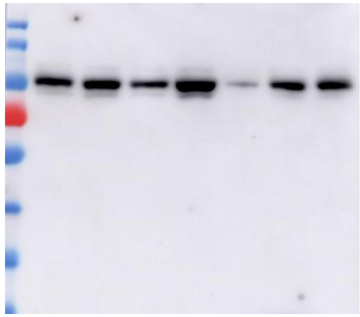

figure 6C
AGS-p100

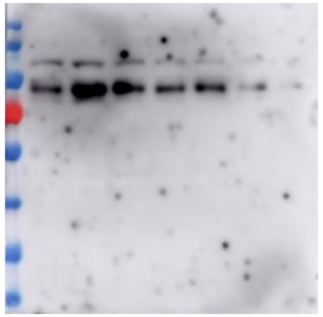

figure 6C
AGS-pCTTN

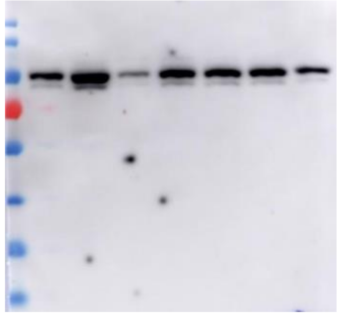

figure 6C
AGS-pSTAT3

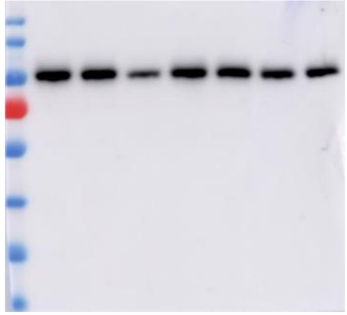

figure 6C
AGS-STAT3

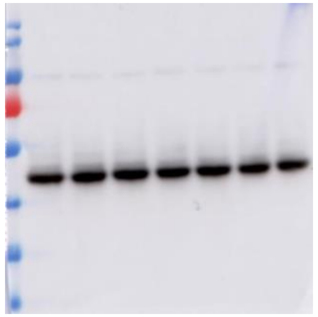

figure 6C
AGS-β-actin

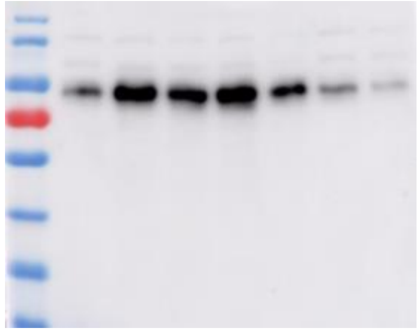

figure 6C
MKN45-CTTN

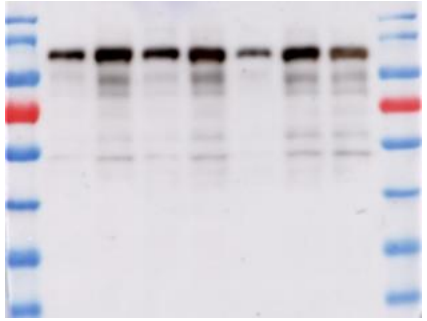

figure 6C
MKN45-p100

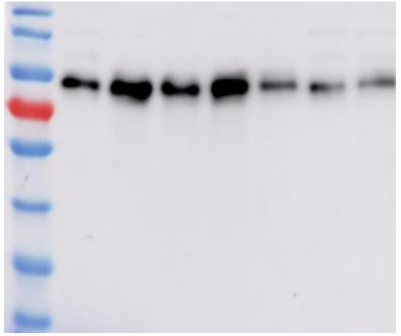

figure 6C
MKN45-pCTTN

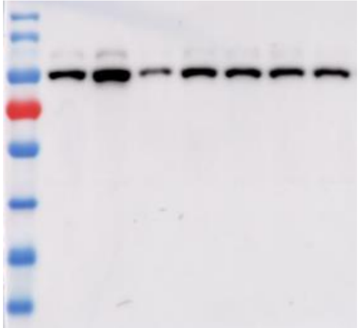

figure 6C
MKN45-pSTAT3

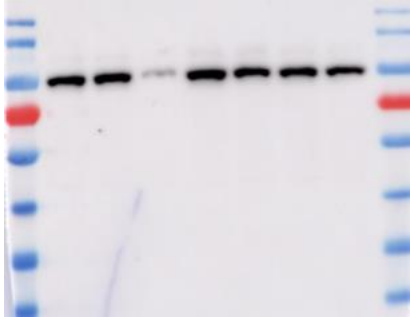

figure 6C
MKN45-STAT3

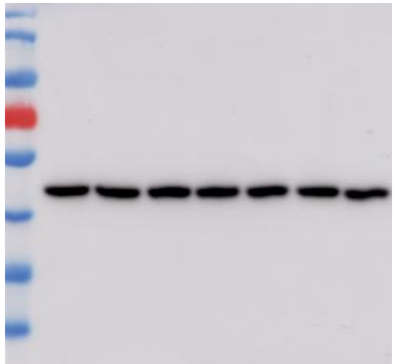

figure 6C
MKN45-β-actin

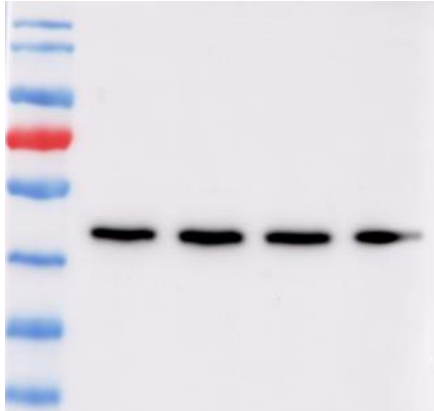

figure S2A β-actin

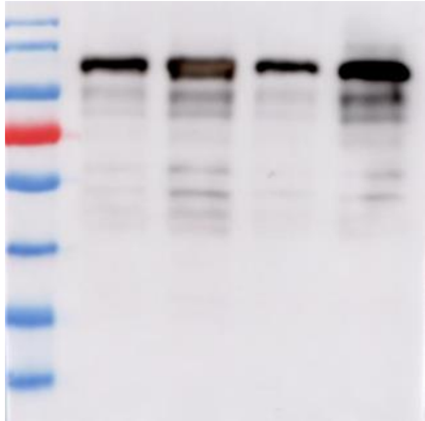

figure S2A TRIM28

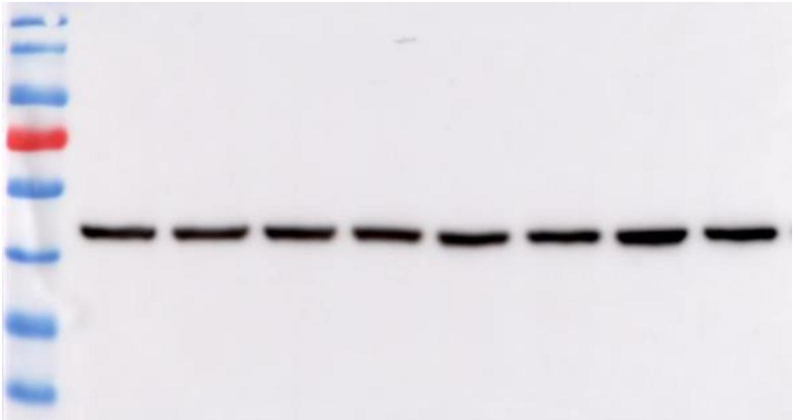

figure S2B β-actin

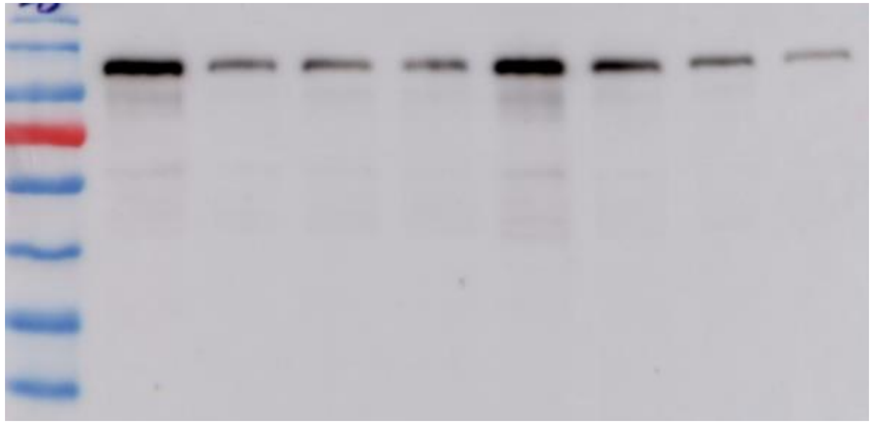

figure S2B TRIM28

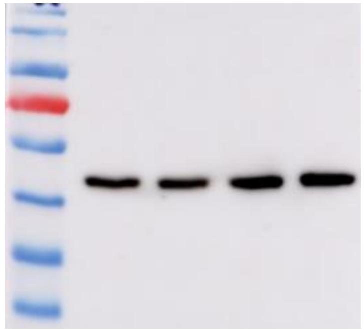

figure S3A β-actin

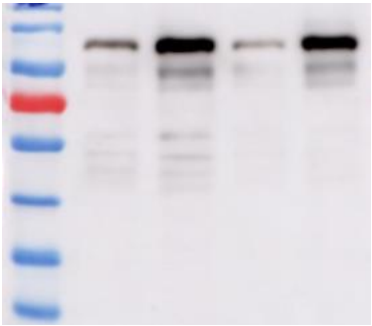

figure S3A CTTN

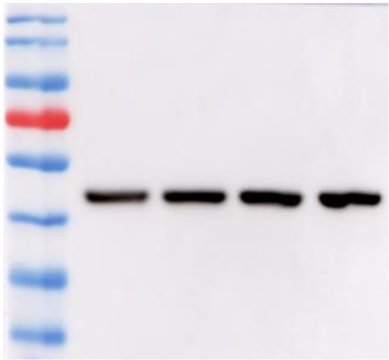

figure S3B MKN45 β-actin

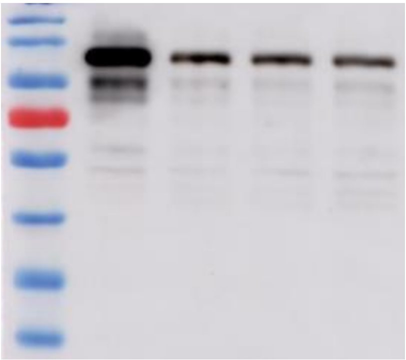

figure S3B MKN45 CTTN

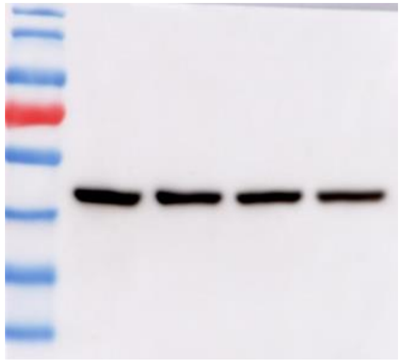

figure S3B AGS β-actin

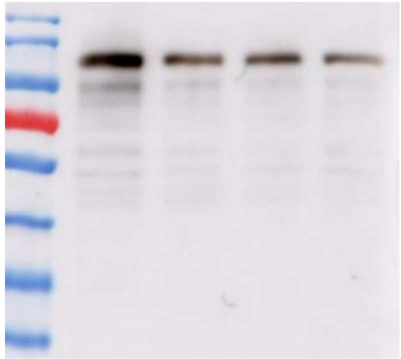

figure S3B AGS CTTN
